# N6-methyladenosine triggers renal fibrosis *via* enhancing translation and stability of *ZEB2* mRNA

**DOI:** 10.1016/j.jbc.2024.107598

**Published:** 2024-07-24

**Authors:** Yating Cai, Jiawang Zhou, Abai Xu, Jinchang Huang, Haisheng Zhang, Guoyou Xie, Ke Zhong, You Wu, Pengfei Ye, Hongsheng Wang, Hongxin Niu

**Affiliations:** 1 Department of Nephrology, Zhujiang Hospital, Southern Medical University, Guangzhou, China; 2 Department of Nephrology, Guangdong Provincial People’s Hospital (Guangdong Academy of Medical Sciences), Southern Medical University, Guangzhou, China; 3 Guangdong Provincial Key Laboratory of New Drug Design and Evaluation, School of Pharmaceutical Sciences, Sun Yat-sen University, Guangzhou, China; 4 Department of Urology, Zhujiang Hospital, Southern Medical University, Guangzhou, China; 5 Department of General Practice, Zhujiang Hospital, Southern Medical University, Guangzhou, China; 6 Special Medical Service Center, Zhujiang Hospital, Southern Medical University, Guangzhou, China

**Keywords:** ZEB2, METTL3, renal fibrosis, translation, mRNA stability

## Abstract

In recent years, a surge in studies investigating N6-methyladenosine (m^6^A) modification in human diseases has occurred. However, the specific roles and mechanisms of m^6^A in kidney disease remain incompletely understood. This study revealed that m^6^A plays a positive role in regulating renal fibrosis (RF) by inducing epithelial-to-mesenchymal phenotypic transition (EMT) in renal tubular cells. Through comprehensive analyses, including m^6^A sequencing, RNA-seq, and functional studies, we confirmed the pivotal involvement of zinc finger E-box binding homeobox 2 (ZEB2) in m^6^A-mediated RF and EMT. Notably, the m^6^A-modified coding sequence of ZEB2 mRNA significantly enhances its translational elongation and mRNA stability by interacting with the YTHDF1/eEF-2 complex and IGF2BP3, respectively. Moreover, targeted demethylation of ZEB2 mRNA using the dm^6^ACRISPR system substantially decreases ZEB2 expression and disrupts the EMT process in renal tubular epithelial cells. *In vivo* and clinical data further support the positive influence of m^6^A/ZEB2 on RF progression. Our findings highlight the m^6^A-mediated regulation of RF through ZEB2, revealing a novel therapeutic target for RF treatment and enhancing our understanding of the impact of mRNA methylation on kidney disease.

Renal fibrosis (RF) represents a prevalent pathological consequence of progressive kidney disease, resulting in compromised kidney function and eventual organ failure (1, 2). Currently, there are no effective treatments for RF, and its incidence is increasing (3). RF involves a diverse range of cells, including tubular cells, peritubular capillary endothelial cells, pericytes, interstitial fibroblasts, and dendritic cells (4). Previously, myofibroblasts received the majority of the attention due to their pivotal role in extracellular matrix synthesis (5). However, new research focuses on how the proximal tubule, a specialized epithelial section susceptible to damage, plays a critical role in RF (6). Renal tubular cells perform the functions of reabsorption, secretion, and excretion (7). Tubular epithelial cells (TECs) are thought to be the major targets and initial reactors after kidney damage due to their high energy requirements and metabolic activity (7). Maladaptive repair of the tubular epithelium is thought to be a critical stage in kidney fibrosis (8). Renal interstitial fibroblasts, which reside in the interstitium and connect the neighboring tubules, provide crucial structural support and allow tissue remodeling by modulating extracellular matrix components. As a result, they are regarded as the ultimate executor in the progression of RF (8). Furthermore, as neighboring cells, TECs and fibroblasts frequently communicate and convey signals to help kidney repair in physiological conditions or exacerbate disease progression in maladaptive situations (8). Renal tubular cells, the predominant and vulnerable intrinsic cells in the kidney, play a pivotal role in maintaining renal function (9). Notably, RF is characterized by the transformation of TECs into mesenchymal-like cells (10, 11), a process known as epithelial-to-mesenchymal transition (EMT). EMT of TECs has been observed in human fibrotic kidneys, and the enrichment of transcription factors associated with EMT is correlated with disease progression (12, 13). Hence, elucidating the mechanisms underlying EMT during RF development and developing targeted and effective treatments are clinically important.

The EMT of TECs is orchestrated by transcriptional regulators such as Snail, zinc finger E-box–binding homeobox 1/2 (ZEB1/2), and Slug (14, 15). Furthermore, epigenetic factors, including DNA methylation, histone modification, and noncoding RNA, have been implicated in orchestrating EMT during RF (16). Inhibition of the enhancer of zeste homolog 2 mitigates RF by preserving smad7 and phosphatase and tensin homolog expression (17). Hypermethylation of KLF4, which is directly mediated by Dnmt1, contributes to EMT progression in renal epithelial cells (18). Moreover, epigenetic factors can modulate the activity of transcriptional regulators of EMT, thereby influencing RF progression. For instance, G9a interacts with Snail, leading to elevated H3K9me2 levels and subsequently reduced E-cadherin expression (19). This suggests that epigenetic regulation is crucial for both EMT and RF progression.

As one of the new emerging epigenetic regulators, RNA modification has attracted increasing attention because of its biological functions. N^6^-methyladenosine (m^6^A) modification is the most abundant in eukaryotic mRNAs (20). m^6^A modification is regulated by the methyltransferase complex, demethylases and RNA-binding proteins, which are dynamic and reversible (21). Methyltransferase-like 3/14 (METTL3/14) and Wilms’ tumor 1–associating protein form the core methyltransferase complex (21). Fat mass and obesity-associated protein (FTO) and AlkB homolog 5 (ALKBH5) have been identified as demethylases (21). m^6^A modifications play an important role in the regulation of major biological behaviors of mRNAs, such as transcription, splicing, translation, intracellular distribution, and degradation (22). Recently, some reports have indicated that m^6^A modification plays a crucial role in a variety of renal diseases, such as renal clear cell carcinoma (23, 24, 25), acute kidney injury (26, 27, 28), diabetic nephropathy (29, 30) and autosomal dominant polycystic kidney disease (31). The roles of RNA m^6^A methylation in modulating RF progression have just recently been investigated. METTL3, the methyltransferase of mRNA m^6^A, demonstrates numerous mechanisms in RF, including: METTL3 regulates the Ena/VASP-like m^6^A alteration, which causes kidney fibrosis (32). METTL3 regulates the WNT1 inducible signaling pathway protein 1 m^6^A alteration, which inhibits the biological functions of high glucose-induced HK2 cells (33). Long noncoding RNA AI662270 induces kidney fibrosis by increasing METTL3-mediated m^6^A alteration of CTGF mRNA (34). METTL3 regulates the NET1 m^6^A alteration, which causes kidney fibrosis (35). Together, these results suggest that m^6^A modification found in mRNA may regulate the progression of RF.

Our study demonstrated that m^6^A positively regulates RF by modulating ZEB2. Specifically, the m^6^A-modified coding sequence (CDS) of *ZEB2* mRNA significantly enhances its translational elongation and mRNA stability by interacting with the YTHDF1/eEF-2 complex and IGF2BP3, respectively. These findings suggest that targeting the m^6^A-mediated regulation of RF through ZEB2 could provide a novel therapeutic avenue for RF treatment.

## Results

### m^6^A was involved in the EMT of renal tubular cells

Transforming growth factor (TGF)-β is considered an efficient inducer of EMT in cells (36). We treated HK-2 or human kidney proximal tubular (HKC-8) cells with 10 ng/ml TGF-β for 3 days, resulting in renal tubular cells exhibiting a mesenchymal morphology characterized by an elongated and spindled morphology (Fig. S1*A*). TGF-β treatment significantly increased the migration of both HK-2 and HKC-8 cells, as previously reported (Fig. S1, *B* and *C*). Additionally, we detected the upregulation of α-smooth muscle actin (α-SMA) and N-Cad mRNA *via* quantitative reverse transcription polymerase chain reaction (qRT-PCR) (Fig. S1*D*). Western blot analysis further confirmed that TGF-β induced the upregulation of α-SMA and N-Cad in HK-2 and HKC-8 cells (Fig. S1, *E* and *F*). These data indicated that renal tubular cells treated with TGF-β underwent EMT.

**Figure 1:**
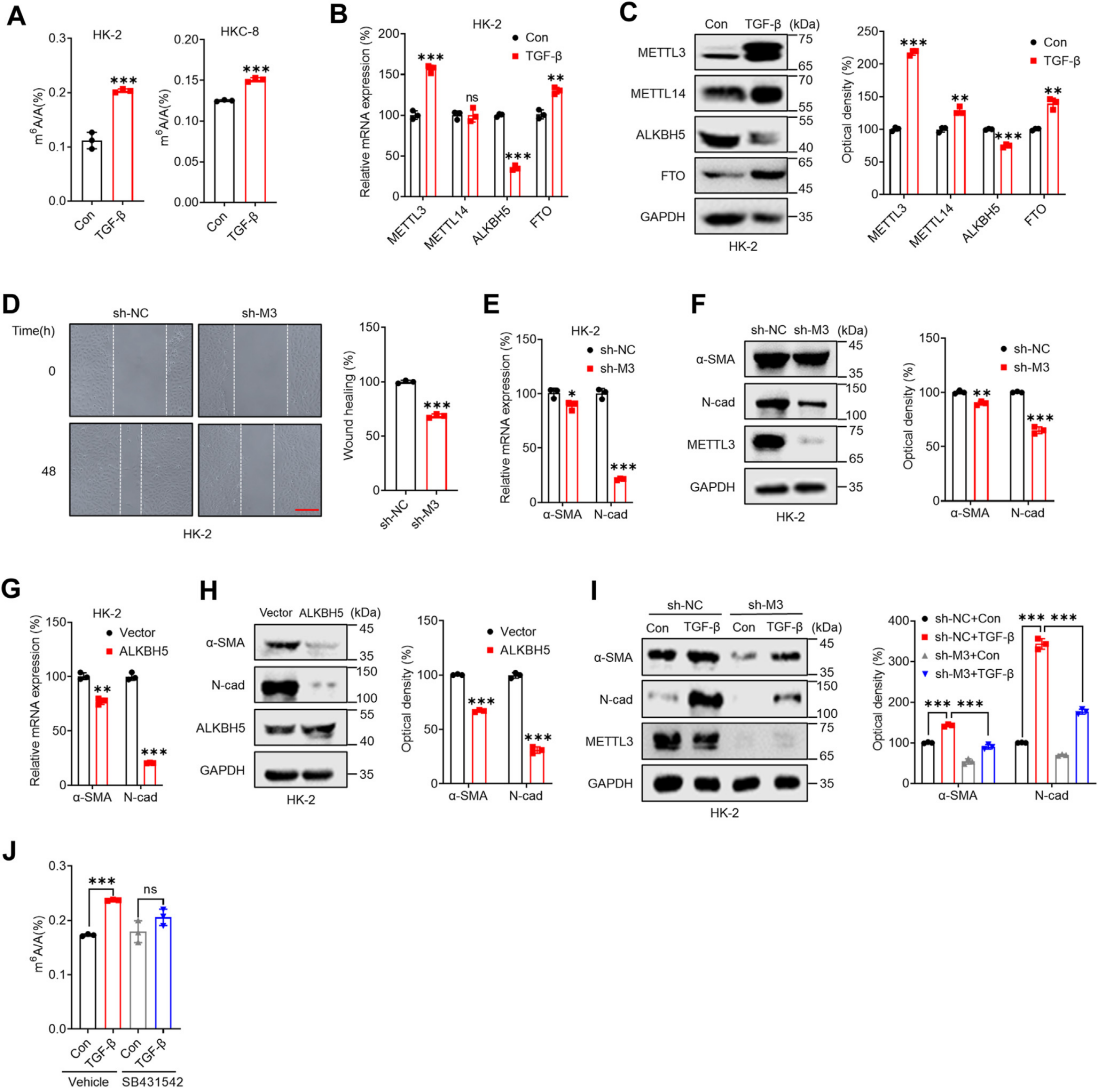
**m**^**6**^**A is involved in the EMT of renal tubular cells.***A*, cells were treated with or without 10 ng/ml TGF-β for 3 days, and the m^6^A/A ratio of the total mRNA was determined by LC-MS/MS. *B*, in HK-2 cells treated with or without 10 ng/ml TGF-β for 24 h, the mRNA levels of m^6^A methyltransferases (*METTL3* and *METTL14*), and m^6^A demethylases (*ALKBH5* and *FTO*) were measured *via* qRT-PCR. *C*, in HK-2 cells treated with or without 10 ng/ml TGF-β for 48 h, the protein levels of m^6^A methyltransferases (METTL3 and METTL14) and m^6^A demethylases (ALKBH5 and FTO) were measured by Western blot analysis (*left*) and quantitatively analyzed (*right*). *D*, wound healing of sh-control or sh-*METTL3* HK-2 cells was recorded (*left*) and quantitatively analyzed (*right*). (The scale bar represents 200 μm). *E*, the mRNA levels of *α-SMA* and *N-Cad* in sh-control or sh-*METTL3* HK-2 cells were measured by qRT-PCR. *F*, the protein levels of α-SMA and N-Cad in sh-control or sh-*METTL3* HK-2 cells were measured by Western blot analysis (*left*) and quantified (*right*). *G*, HK-2 cells were transfected with pcDNA (vector) or pcDNA/ALKBH5 for 24 h, and the mRNA levels of *α-SMA* and *N-Cad* were measured by qRT-PCR. *H*, HK-2 cells were transfected with pcDNA (vector) or pcDNA/ALKBH5 for 48 h, and the protein expression levels of α-SMA and N-Cad were measured by Western blot analysis (*left*) and quantified (*right*). *I*, sh-control or sh-*METTL3* HK-2 cells were treated with or without 10 ng/ml TGF-β for 3 days, and the protein levels of α-SMA and N-Cad were measured by Western blot analysis (*left*) and quantitatively analyzed (*right*). *J*, HK-2 cells were pretreated with or without the Smad2/3 inhibitor SB431542 (10 μM) and then further treated with 10 ng/ml TGF-β for 3 days. The m^6^A/A ratio of the total mRNA was determined by LC-MS/MS. The data are presented as the means ± SDs from three independent experiments. ∗*p* < 0.05, ∗∗*p* < 0.01, ∗∗∗*p* < 0.001, NS, not significant; and Student’s *t* test. α-SMA, α-smooth muscle actin; ALKBH5, AlkB homolog 5; EMT, epithelial-to-mesenchymal phenotypic transition; FTO, fat mass and obesity-associated protein; m^6^A, N6-methyladenosine; METTL3, methyltransferase-like 3; TGF, transforming growth factor.

To verify whether m^6^A was involved in EMT in renal tubular cells, we examined the variations in m^6^A levels in the relevant cells. LC-MS/MS analysis indicated that the m^6^A levels of mRNAs isolated from HK-2 or HKC-8 cells treated with TGF-β were significantly greater than those of their corresponding control cells (Fig. 1*A*). This finding was further confirmed by the results of dot-blot analysis (Fig. S1*G*), which indicated that renal tubular cells undergoing EMT exhibited increased m^6^A mRNA levels.

Since m^6^A was increased in renal tubular cells undergoing EMT, we further investigated the variations in m^6^A methyltransferases (METTL3 and METTL14) and demethylases (FTO and ALKBH5). qRT-PCR analysis revealed that the expression of METTL3 was upregulated while that of ALKBH5 was decreased in both HK-2 and HKC-8 cells treated with TGF-β (Figs. 1*B* and S2*A*). Consistently, Western blot analysis confirmed the upregulation of METTL3 and downregulation of ALKBH5 in renal tubular cells undergoing EMT (Figs. 1*C* and S2*B*). To understand the role of m^6^A in the EMT process, we used sh-*METTL3* to knock down METTL3 in HK-2 and HKC-8 cells (Fig. S2*C*). LC-MS/MS confirmed that sh-*METTL3* cells had significantly lower levels of m^6^A than control cells (Fig. S2*D*). METTL3 knockdown significantly decreased the migration of both HK-2 (Fig. 1*D*) and HKC-8 (Fig. S2*E*) cells. Furthermore, the mRNA (Figs. 1*E* and S2*F*) and protein (Figs. 1*F* and S2*G*) levels of both α-SMA and N-Cad were decreased in sh-*METTL3* cells.

We undertook further investigations to explore the potential role of ALKBH5 in the EMT process. Our results revealed that the overexpression of ALKBH5 led to a decrease in the mRNA levels of α-SMA and N-Cad in HK-2 and HKC-8 cells (Figs. 1*G* and S2*H*). Moreover, our Western blot analysis corroborated these observations by demonstrating that ALKBH5 overexpression decreased the levels of α-SMA and N-Cad in HK-2 and HKC-8 cells (Figs. 1*H* and S2*I*). These findings were further supported by the results obtained for si-METTL3 (as shown in Fig. S3, *A* and *E*) and the METTL3 inhibitor STM2457 (as shown in Fig. S3, *B* and *F*). Collectively, these results indicate that cellular m^6^A plays a significant role in regulating the EMT process in renal tubular cells.

Furthermore, whether m^6^A is involved in TGF-β-induced EMT was investigated. Our data showed that the TGF-β–induced upregulation of α-SMA and N-Cad in renal tubular cells was reversed in sh-*METTL3* cells (Figs. 1*I* and S2*J*). Consistently, both si-METTL3 (Fig. S3, *C* and *G*) and the METTL3 inhibitor STM2457 (Fig. S3, *D* and *H*) rescued the TGF-β–induced EMT potential of renal tubular cells. Previous studies have demonstrated that in human embryonic stem cells, TGF-β can induce mRNA methylation by interacting with Smad2/3 and the METTL3–METTL14–Wilms’ tumor 1–associating protein complex (37). LC-MS/MS analysis revealed that the Smad2/3 inhibitor SB431542 prevented the increase in m^6^A in TGF-β–treated HK-2 cells (Fig. 1*J*). Collectively, these findings indicated that the modulation of m^6^A levels in mRNA governs the EMT process in renal tubular cells.

### ZEB2 was involved in m^6^A-regulated EMT in renal tubular cells

**Figure 2:**
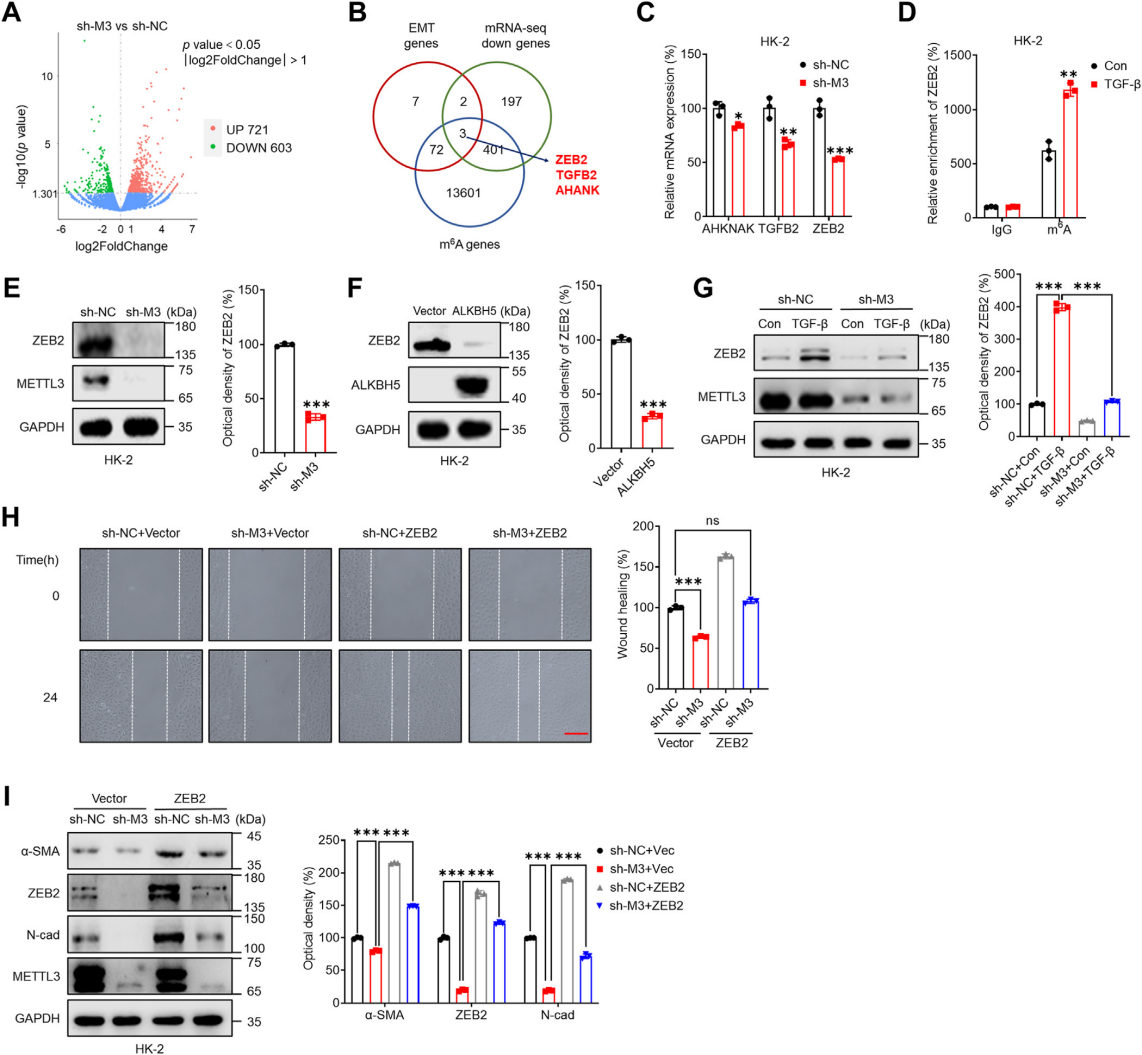
**ZEB2 was involved in m**^**6**^**A-regulated EMT in renal tubular epithelial cells.***A*, volcano plot of RNA-seq analysis showing differentially expressed genes between sh-control and sh-*METTL3* HK-2 cells. *B*, Venn diagram showing substantial and significant overlap among EMT genes, downregulated genes in sh-*METTL3* HK-2 cells (>2-fold), and m^6^A-enriched genes in the kidneys of WT mice. *C*, the mRNA levels of *AHNAK, TGFB2,* and *ZEB2* in sh-control or sh-*METTL3* HK-2 cells were measured by qRT-PCR. *D*, m^6^A RIP-qPCR analysis of *ZEB2* mRNA in control and EMT HK-2 cells. *E*, the protein levels of ZEB2 in sh-control or sh-*METTL3* HK-2 cells were determined by Western blot analysis (*left*) and quantified (*right*). *F*, HK-2 cells were transfected with pcDNA (vector) or pcDNA/ALKBH5 for 48 h, and the protein expression of ZEB2 was measured by Western blot analysis (*left*) and quantified (*right*). *G*, sh-control or sh-*METTL3* HK-2 cells were treated with or without 10 ng/ml TGF-β for 48 h, and the protein levels of ZEB2 were determined by Western blot analysis (*left*) and quantified (*right*). *H*, the extent of wound healing in sh-control or sh-*METTL3* HK-2 cells transfected with or without pcDNA/ZEB2 for 48 h was recorded (*left*), and the data were quantitatively analyzed (*right*). (The scale bar represents 200 μm). *I*, sh-control or sh-*METTL3* HK-2 cells were transfected with or without pcDNA/ZEB2 for 48 h, and the protein levels of ZEB2, α-SMA, and N-Cad were measured by Western blot analysis (*left*) and quantitatively analyzed (*right*). The data are presented as the means ± SDs from three independent experiments. ∗*p <* 0.05, ∗∗*p <* 0.01, ∗∗∗*p <* 0.001, NS, not significant; and Student’s *t* test. α-SMA, α-smooth muscle actin; ALKBH5, AlkB homolog 5; EMT, epithelial-to-mesenchymal phenotypic transition; m^6^A, N6-methyladenosine; METTL3, methyltransferase-like 3; RIP, RNA binding protein immunoprecipitation; TGF, Transforming growth factor; ZEB2, zinc finger E-box–binding homeobox 2.

We proceeded to study potential targets implicated in m^6^A-regulated EMT in renal tubular cells using mRNA-seq and m^6^A sequencing (m^6^A-seq) data (38). According to the RNA-seq analysis, 603 genes were downregulated and 721 genes were upregulated in sh-*METTL3* HK-2 cells compared with sh-control cells (Fig. 2*A*). Among the 84 EMT-related genes (Table S5), three candidates, AHNAK, TGFB2, and ZEB2, that overlapped among various genes according to mRNA-seq (greater than 2.0-fold variation, *p* < 0.05) between sh-control and sh-*METTL3* HK-2 cells and were modified by m^6^A in the kidney based on m^6^A-seq data (Fig. 2*B*). The qRT-PCR results confirmed the decreased expression of three candidate genes in both sh-*METTL3* HK-2 (Fig. 2*C*) and sh-*METTL3* HKC-8 (Fig. S4*A*) cells, which was consistent with the mRNA-seq data. Since ZEB2 has been reported to be one of the most important regulatory factors for EMT (35) and is the most downregulated gene in sh-*METTL3* cells, we focused on whether ZEB2 is involved in the m^6^A-regulated EMT of renal tubular cells.

By m^6^A-RNA binding protein immunoprecipitation (RIP)-qPCR, we confirmed that the m^6^A antibody enriched *ZEB2* mRNA more than 5-fold in HK-2 cells. The m^6^A level of *ZEB2* mRNA significantly increased in renal tubular cells undergoing EMT, and the relative enrichment of m^6^A was 1.7-fold greater in these cells than in control cells (Fig. 2*D*). Knockdown of METTL3 (Figs. 2*E* and S4*B*) and overexpression of ALKBH5 (Figs. 2*F* and S4*C*) markedly inhibited the protein expression of ZEB2 in renal tubular cells. Both si-METTL3 (Fig. S4*D*) and the METTL3 inhibitor STM2457 (Fig. S4*E*) decreased the protein expression of ZEB2 in renal tubular cells. To confirm the effect of m^6^A on ZEB2 expression, we treated sh-control and sh-*METTL3* cells with TGF-β. The results showed that the TGF-β–induced expression of ZEB2 was reduced in sh-*METTL3* HK-2 (Fig. 2*G*) and sh-*METTL3* HKC-8 (Fig. S4*F*) cells. Both si-METTL3 (Fig. S4*G*) and the METTL3 inhibitor STM2457 (Fig. S4*H*) attenuated the TGF-β–induced expression of ZEB2 in renal tubular cells, indicating that METTL3 mediates the TGF-β–induced expression of ZEB2 in renal tubular cells.

Although ZEB2 has been reported to be a key regulatory factor in EMT (39), we further confirmed whether ZEB2 was involved in the m^6^A-induced EMT of tubular cells. Our wound healing data showed that overexpression of ZEB2 attenuated the suppression of cell migration *via* METTL3 knockdown in HK-2 cells (Fig. 2*H*). Similarly, overexpression of ZEB2 reversed the downregulation of the mesenchymal markers α-SMA and N-Cad (Figs. 2*I* and S4*I*) in sh-*METTL3* HK-2 and sh-*METTL3* HKC-8 cells. These data indicated that ZEB2 was involved in m^6^A-regulated EMT in renal TECs.

### m^6^A regulated the mRNA stability and translation of ZEB2 mRNA

**Figure 3:**
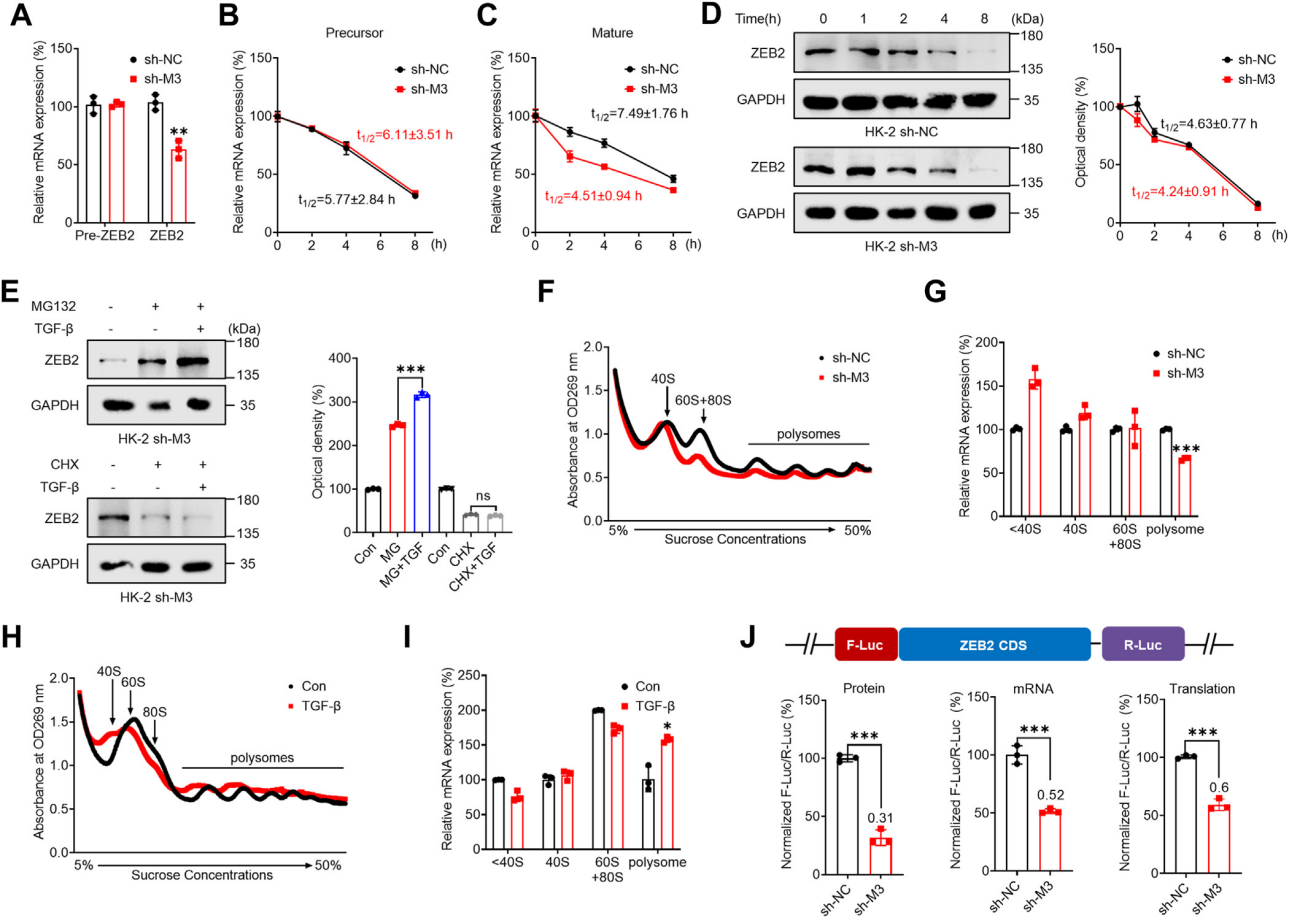
**m**^**6**^**A regulated *ZEB2* mRNA stability and translation in renal tubular epithelial cells.***A*, the levels of the precursor and mature mRNA of *ZEB2* in sh-control or sh-*METTL3* HK-2 cells were measured *via* qRT-PCR. *B*, sh-control or sh-*METTL3* HK-2 cells were pretreated with Act-D for the indicated times, and the mRNA levels of the precursor *ZEB2* were analyzed at the indicated times. *C*, sh-control or sh-*METTL3* HK-2 cells were pretreated with Act-D for the indicated times, and mature *ZEB2* mRNA was analyzed at the indicated times. *D*, sh-control or sh-*METTL3* HK-2 cells were treated with CHX for the indicated times, and the protein expression of ZEB2 was analyzed by Western blot analysis (*left*) and quantitative analysis (*right*). *E*, sh-*METTL3* HK-2 cells were pretreated with CHX or MG-132 for 6 h and then further treated with or without 10 ng/ml TGF-β for 48 h. ZEB2 expression was detected by Western blot analysis (*left*) and quantitatively analyzed (*right*). *F*, polysome profiling of sh-control or sh-*METTL3* HK-2 cells was performed. *G*, analysis of *ZEB2* mRNA in the nonribosome (<40S), 40S, 60S, 80S, and polysome fractions of sh-*METTL3* HK-2 cells compared to sh-control HK-2 cells. *H*, polysome profiling of HK-2 cells treated with or without 10 ng/ml TGF-β for 3 days. *I*, analysis of *ZEB2* mRNA in the nonribosome (<40 S), 40 S, 60 S, 80 S, and polysome fractions of HK-2 cells undergoing EMT compared with that in HK-2 WT cells. *J*, sh-control or sh-*METTL3* HK-2 cells were transfected with the pmirGLO-ZEB2 reporter for 24 h, and the translation efficiency of ZEB2 was defined as the quotient of reporter protein production (F-luc/R-luc) divided by mRNA abundance. The data are presented as the means ± SDs from three independent experiments. ∗*p <* 0.05, ∗∗*p <* 0.01, ∗∗∗*p <* 0.001, NS, not significant; and Student’s *t* test. CHX, cycloheximide; EMT, epithelial-to-mesenchymal phenotypic transition; F-luc, firefly luciferase; m^6^A, N6-methyladenosine; METTL, methyltransferase-like 3; R-luc, renilla luciferase; TGF, transforming growth factor; ZEB2, zinc finger E-box–binding homeobox 2.

Our investigation delved deeper into the mechanism underlying m^6^A the regulation of ZEB2 expression by m^6^A. Initially, we analyzed the expression of the precursor (pre) and maturation (mat-) mRNAs of *ZEB2*. Our results revealed no significant difference in the expression of the precursor mRNA of *ZEB2* between sh-*METTL3* and sh-control HK-2 cells. However, we observed a significant decrease in the expression of the mature *ZEB2* mRNA in sh-*METTL3* HK-2 cells (Fig. 3*A*). Similar results were obtained in HKC-8 cells (Fig. S5*A*). To determine the effect of m^6^A on the stability of *ZEB2* mRNA, we treated sh-control and sh-*METTL3* HK-2 cells with actinomycin-D (Act-D) to block transcription. Our results showed that METTL3 had no significant impact on the stability of precursor *ZEB2* mRNA in HK-2 cells, indicating that m^6^A does not affect the splicing rate of precursor *ZEB2* mRNA (Fig. 3*B*). However, in sh-*METTL3* HK-2 cells, we observed a significant reduction in the mRNA stability of mature *ZEB2* (Fig. 3*C*). Similar results were also obtained in HKC-8 cells (Fig. S5, *B* and *C*). Our fractionation-qPCR results indicated no difference in the subcellular localization of *ZEB2* mRNA between sh-control and sh-*METTL3* HK-2 cells (Fig. S5*D*). Collectively, these results suggest that METTL3 increases the mRNA stability of *ZEB2* in renal tubular cells.

We conducted further investigations to determine whether m^6^A can regulate ZEB2 expression through means other than mRNA stability. We treated sh-control and sh-*METTL3* HK-2 cells with the protein translation inhibitor cycloheximide (CHX) and found no significant difference in the ZEB2 protein half-life between the two cell lines. This indicated that m^6^A-regulated ZEB2 expression is not related to protein stability (Fig. 3*D*). We also pretreated sh-*METTL3* HK-2 cells with MG-132 to inhibit proteasome activity or with CHX to block protein translation, followed by treatment with TGF-β for 3 days. Our data showed that CHX, but not MG132, attenuated the induction of ZEB2 by TGF-β in sh-*METTL3* HK-2 cells (Fig. 3*E*). This suggested that the m^6^A-induced expression of ZEB2 may be associated with translation.

To investigate this further, we separated RNA fractions from sh-control and sh-*METTL3* HK-2 cells using ribosome profiling (Fig. 3*F*). Our results showed that *ZEB2* mRNA in translation-active polysomes (>80 S) was significantly lower in sh-*METTL3* HK-2 cells than in sh-control HK-2 cells (Fig. 3*G*), suggesting that m^6^A may regulate the translation elongation of ZEB2. We applied the same approaches in EMT cells and found that *ZEB2* mRNA in translation-active polysomes (>80 S) was significantly greater in renal tubular cells undergoing EMT than in control cells (Fig. 3, *H* and *I*). Additionally, we constructed a pmirGLO-ZEB2 luciferase reporter by fusing ZEB2 complementary DNA to firefly luciferase (F-luc). Our data showed that the translation efficiency of ZEB2 in sh-*METTL3* HK-2 cells was significantly lower than that in sh-control HK-2 cells (Fig. 3*J*). These data confirmed that METTL3 can trigger the translation of ZEB2 in renal tubular cells.

We also investigated whether m^6^A affects ZEB2 translation through cap-dependent or non-cap-dependent translation. We treated sh-control and sh-*METTL3* HK-2 cells with rapamycin (40) and found no significant difference in ZEB2 levels between the two cell lines. However, cotreatment with the protein synthesis inhibitor CHX and rapamycin resulted in a rapid decrease in ZEB2 levels in sh-control HK-2 cells (Fig. S5*E*), suggesting that ZEB2 expression is regulated through cap-independent translation.

### Methylation sites mediate m^6^A-regulated ZEB2

**Figure 4:**
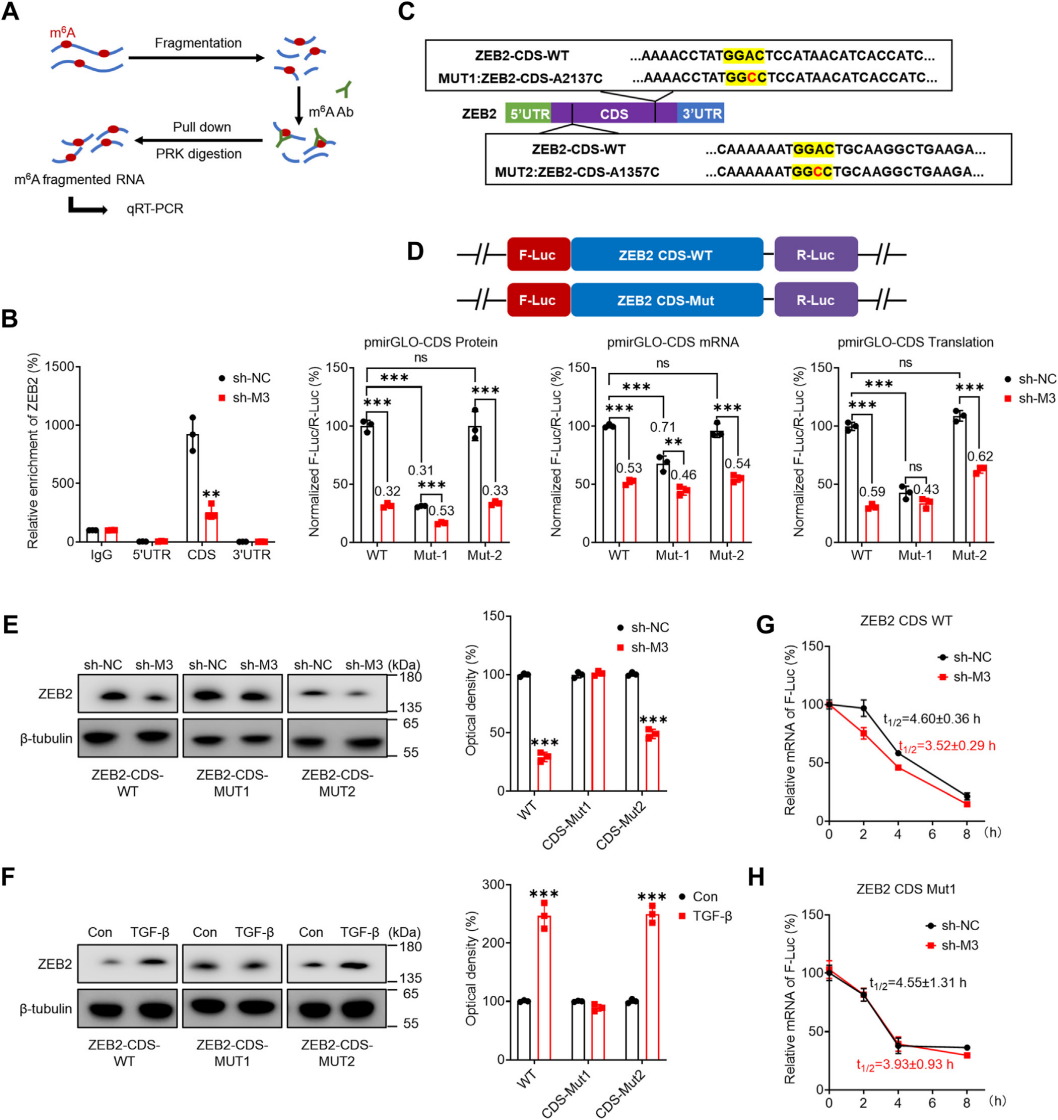
**Methylation sites of ZEB2 involved in m**^**6**^**A-mediated regulation of ZEB2 expression.***A*, schematic representation of m^6^A RIP-PCR with fragmented RNA from cells. *B*, m^6^A RIP-qPCR analysis of *ZEB2* mRNA in sh-control or sh-*METTL3* HK-2 cells by using fragmented RNA. *C*, schematic representation of mutations in the CDS to investigate the role of m^6^A in ZEB2 expression. *D*, sh-control or sh-*METTL3* HK-2 cells were transfected with pmirGLO-ZEB2-CDS-WT or pmirGLO-ZEB2-CDS-Mut1/2 reporters for 24 h. The protein, mRNA, and translation efficiencies were determined. *E*, pcDNA-ZEB2-CDS-WT or pcDNA-ZEB2-CDS-Mut1/2 was transfected into sh-control or sh-*METTL3* HK-2 cells for 48 h, and the protein levels of ZEB2 were determined by Western blot analysis (*left*) and quantitatively analyzed (*right*). *F*, after transfection with pcDNA-ZEB2-CDS-WT or pcDNA-ZEB2-Mut1/2, HK-2 cells were further treated with or without 10 ng/ml TGF-β for 48 h, and the protein levels of ZEB2 were determined by Western blot analysis (*left*) and quantitatively analyzed (*right*). *G*, pcDNA-ZEB2-CDS-WT was transfected into sh-control or sh-*METTL3* HK-2 cells for 24 h, and the cells were then further treated with Act-D for the indicated times. The mRNA level of *ZEB2* was determined by qRT-PCR. *H*, pcDNA-ZEB2-CDS-Mut1 was transfected into sh-control or sh-*METTL3* HK-2 cells for 24 h, and the cells were then further treated with Act-D for the indicated times. The mRNA level of *ZEB2* was determined by qRT-PCR. The data are presented as the means ± SDs from three independent experiments. ∗*p <* 0.05, ∗∗*p <* 0.01, ∗∗∗*p <* 0.001, NS, not significant; and Student’s *t* test. Act-D, actinomycin-D; CDS, coding sequence; m^6^A, N6-methyladenosine; METTL3/14, methyltransferase-like 3/14; RIP, RNA binding protein immunoprecipitation; TGF, transforming growth factor; ZEB2, zinc finger E-box–binding homeobox 2.

To characterize m^6^A methylation in *ZEB2* mRNA, fragmented RNA isolated from sh-control or sh-*METTL3* HK-2 cells was immunoprecipitated with a m^6^A antibody (Fig. 4*A*). m^6^A-RIP-PCR showed that the m^6^A enrichment in the *ZEB2* mRNA CDS was greater than that in the 5′UTR and 3′UTR. Moreover, this enrichment was considerably decreased in sh-*METTL3* HK-2 cells (Fig. 4*B*), suggesting that m^6^A methylation in the CDS region might be more dynamic than that in the 5′UTR and 3′UTR. m^6^A-seq and motif analysis revealed two potential m^6^A sites (A1357 and A2137) in the CDS region of *ZEB2* mRNA (Fig. 4*C*). To explore the possible impact of m^6^A methylation sites on ZEB2 expression, CDS reporters containing the WT ZEB2 CDS or mutant 1/2 CDS (GGAC to GGCC) were constructed after the F-luc reporter gene (Fig. 4*D*). Luciferase assays indicated that the mRNA expression, protein expression, and translation efficiency of pmirGLO-ZEB2-CDS in sh-*METTL3* HK-2 cells were significantly lower than those in sh-control HK-2 cells (Fig. 4*D*). Mutation of A2137 (CDS-Mut1, A2137C), but not A1357 (CDS-Mut2, A1357C), in the CDS resulted in decreased mRNA expression, protein expression, and translation efficiency of F-Luc but partially abolished the difference between sh-control and sh-*METTL3* HK-2 cells (Fig. 4*D*).

Furthermore, sh-control or sh-*METTL3* HK-2 cells were further transfected with pcDNA-ZEB2-CDS-WT or pcDNA-ZEB2-CDS-Mut1/Mut2. Western blot analysis revealed that compared with pcDNA-ZEB2-CDS-WT or pcDNA-ZEB2-CDS-Mut2, pcDNA-ZEB2-CDS-CDS-CDS-Mut1 attenuated the METTL3-mediated suppression of ZEB2 expression (Fig. 4*E*). Next, HK-2 WT cells were transfected with pcDNA-ZEB2-CDS-WT or pcDNA-ZEB2-CDS-Mut1/Mut2 and further treated with TGF-β. The Western blot results demonstrated that TGF-β induced lower ZEB2 expression in the pcDNA-ZEB2-CDS-Mut1 group than in the pcDNA-ZEB2-CDS-WT or pcDNA-ZEB2-CDS-mut2 group (Fig. 4*F*). Furthermore, the mRNA stability of pcDNA-ZEB2-5-CDS-WT was greater in sh-control HK-2 cells than in sh-*METTL3* HK-2 cells (Fig. 4*G*), while pcDNA-ZEB2-CDS-Mut1 reversed the difference in the mRNA half-life between sh-control and sh-*METTL3* HK-2 cells (Fig. 4*H*). In summary, our findings suggested that methylation of A2137 within the *ZEB2* mRNA CDS is responsible for mRNA stability and translation.

### Factors involved in the m^6^A-mediated regulation of ZEB2 expression

**Figure 5:**
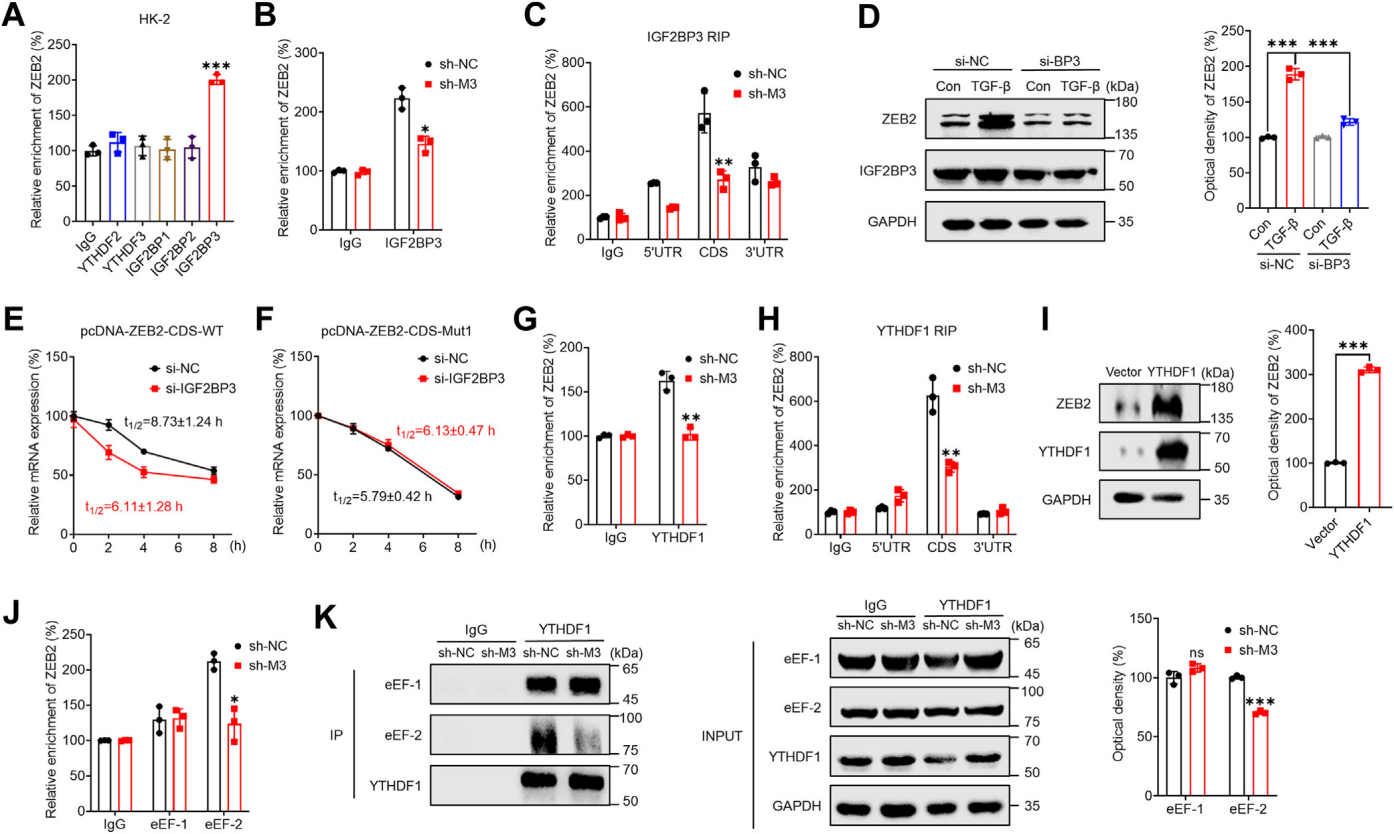
**Factors involved in the m**^**6**^**A-mediated regulation of ZEB2 expression.***A*, RIP-qPCR analysis of *ZEB2* mRNA in HK-2 cells with antibodies against YTHDF2, YTHDF3, and IGF2BP1∼3. *B*, IGF2BP3 RIP-qPCR analysis of *ZEB2* mRNA in sh-control or sh-*METTL3* HK-2 cells. *C*, the binding of IGF2BP3 to the CDS, 5′UTR, and 3′UTR of *ZEB2* mRNA in sh-control or sh-*METTL3* HK-2 cells was analyzed by IGF2BP3 RIP-qPCR using fragmented RNA. *D*, after pretransfection with si-control (si-NC) or si-IGF2BP3 (si-BP3) for 12 h, HK-2 cells were further treated with or without 10 ng/ml TGF-β for 48 h, and the expression of ZEB2 was assessed by Western blot analysis (*left*) and quantitatively analyzed (*right*). *E*, HK-2 cells were transfected with si-control, si-IGF2BP3, or pcDNA-ZEB2-CDS-WT for 24 h and then further treated with Act-D for the indicated times. The mRNA level of *ZEB2* was determined by qRT‒PCR. *F*, HK-2 cells were transfected with si-control, si-IGF2BP3 or pcDNA-ZEB2-CDS-Mut1 for 24 h and then further treated with Act-D for the indicated times. The mRNA level of *ZEB2* was determined by qRT-PCR. *G*, YTHDF1 RIP-qPCR analysis of *ZEB2* mRNA in sh-control or sh-*METTL3* HK-2 cells. *H*, the binding of YTHDF1 to the CDS, 5′UTR, and 3′UTR of *ZEB2* mRNA in sh-control or sh-*METTL3* HK-2 cells was analyzed by YTHDF1 RIP-qPCR using fragmented RNA. *I*, HK-2 cells were transfected with the vector or the YTHDF1 construct for 48 h, and the expression of ZEB2 was determined by Western blot analysis (*left*) and quantitative analysis (*right*). *J*, eEF-1 and eEF-2 RIP-qPCR analysis of *ZEB2* mRNA in sh-control or sh-*METTL3* HK-2 cells. *K*, the binding of YTHDF1 to eEF-1 or eEF-2 in sh-control or sh-*METTL3* HK-2 cells was evaluated by immunoprecipitation (*left*) and quantitative analysis (*right*). The data are presented as the means ± SDs from three independent experiments. ∗*p <* 0.05, ∗∗*p <* 0.01, ∗∗∗*p <* 0.001, NS, not significant; and Student’s *t* test. Act-D, actinomycin-D; ALKBH5, AlkB homolog 5; CDS, coding sequence; EMT, epithelial-to-mesenchymal phenotypic transition; m^6^A, N6-methyladenosine; METTL3, methyltransferase-like 3; RIP, RNA binding protein immunoprecipitation; TGF, transforming growth factor; ZEB2, zinc finger E-box–binding homeobox 2.

We further investigated the mechanisms responsible for the m^6^A-mediated regulation of mRNA stability and translation elongation. Specifically, m^6^A modification can affect mRNA stability through readers such as YTHDF2, YTHDF3, and IGF2BPs (41, 42). Further analysis using RIP-PCR revealed that the antibodies against IGF2BP3, but not YTHDF2, YTHDF3, IGF2BP1, or IGF2BP2, significantly enriched *ZEB2* mRNA in HK-2 cells (Fig. 5*A*). The binding between IGF2BP3 and *ZEB2* mRNA was also decreased in sh-*METTL3* cells (Fig. 5*B*). Additionally, RIP-PCR showed that m^6^A enrichment in the ZEB2 mRNA CDS was significantly enriched by the IGF2BP3 antibody, which was further downregulated in sh-*METTL3* HK-2 cells (Fig. 5*C*). These findings suggested that IGF2BP3 can bind to m^6^A-modified *ZEB2* mRNA in renal tubular cells.

To assess the impact of IGF2BP3 on ZEB2 expression, we overexpressed IGF2BP3 in HK-2 cells, which led to increased ZEB2 protein expression (Fig. S6*A*). Conversely, knockdown of IGF2BP3 attenuated the TGF-β–induced expression of ZEB2 in HK-2 cells (Fig. 5*D*). Moreover, si-IGF2BP3 significantly reduced the mRNA stability of pcDNA-ZEB2-CDS-WT (Fig. 5*E*), while this effect was attenuated for pcDNA-ZEB2-CDS-Mut1 (Fig. 5*F*). Taken together, these results indicated that IGF2BP3 is involved in m^6^A-mediated regulation of mRNA stability. However, the data also showed that IGF2BP3 had no significant effect on the endogenous translation efficiency of ZEB2 or pmirGLO-ZEB2-CDS-WT (Fig. S6, *B* and *C*), suggesting that IGF2BP3 does not contribute to the m^6^A-mediated regulation of ZEB2 translation.

YTHDF1 is capable of recognizing m^6^A-methylated mRNAs and facilitating the translation of their targets (43). According to the RIP-PCR analysis, YTHDF1 significantly interacted with *ZEB2* mRNA, which was inhibited in sh-*METTL3* HK-2 cells (Fig. 5*G*). Additionally, it was discovered that YTHDF1 primarily interacts with the CDS region of *ZEB2* mRNA, as opposed to the 3′UTR or 5′UTR regions (Fig. 5*H*). In sh-*METTL3* HK-2 cells, the interaction between YTHDF1 and the CDS of *ZEB2* mRNA was substantially decreased, while that between YTHDF1 and the 3′UTR or 5′UTR was not (Fig. 5*H*). Furthermore, we knocked down the expression of YTHDF1 in HK-2 cells and observed that si-YTHDF1 mitigated the TGF-β–induced expression of ZEB2 in HK-2 cells (Fig. S6*D*). Overexpression of YTHDF1 increased the protein expression of ZEB2 in HK-2 cells (Fig. 5*I*) but had no effect on the mRNA expression (Fig. S6, *E* and *F*) or stability (Fig. S6, *G*–*J*) of ZEB2 in renal tubular cells. Moreover, overexpression of YTHDF1 increased the protein expression of pcDNA-ZEB2-CDS-WT, while this effect was diminished for pcDNA-ZEB2-CDS-Mut1 (Fig. S6*K*). These findings suggested that YTHDF1 regulated the m^6^A-mediated translation of ZEB2.

We then further evaluated the role of translation elongation in m^6^A-regulated ZEB2 *via* ribosome profiling *via* qRT-PCR. eEF-1 and eEF-2 are elongation factors that regulate eukaryotic translational elongation (44). RIP-qPCR was used to detect the variation in eEF-1 and eEF-2 binding to *ZEB2* mRNA. The results showed that eEF-2 can bind strongly to *ZEB2* mRNA, while this binding was inhibited in sh-*METTL3* HK-2 cells (Fig. 5*J*). Furthermore, co-immunoprecipitation analysis revealed that both eEF-1 and eEF-2 can bind to YTHDF1 in HK-2 cells; however, only the binding of eEF-2 was significantly decreased in sh-*METTL3* HK-2 cells (Fig. 5*K*). These findings suggested that YTHDF1 and eEF-2, but not eEF-1, may be involved in regulating the m^6^A-mediated translation elongation of *ZEB2* mRNA in renal tubular cells.

### Targeting m^6^A of ZEB2 mRNA with m^6^A *via* the dm^6^ACRISPR system to regulate EMT in renal tubular cells

**Figure 6:**
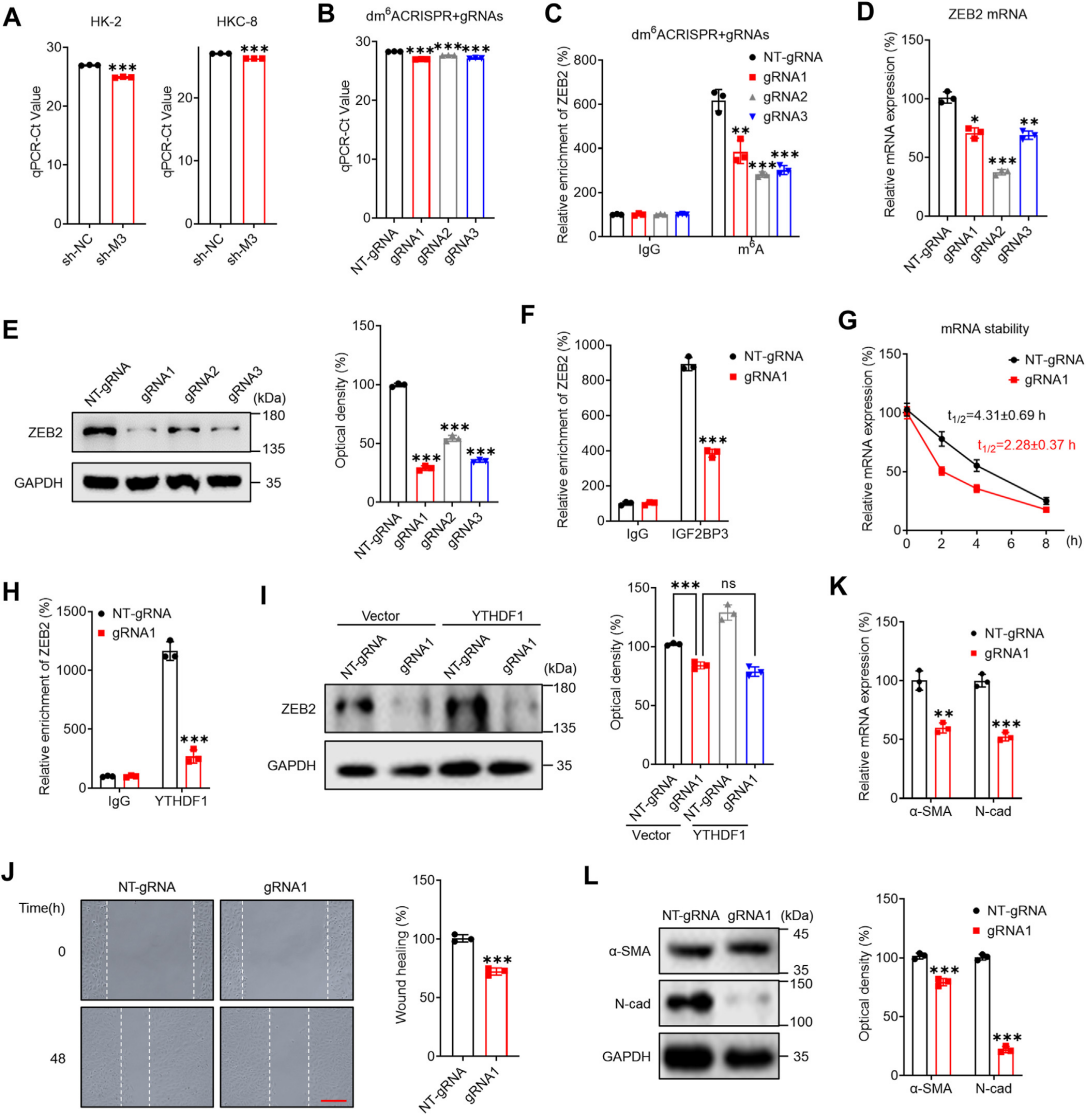
**Targeting m**^**6**^**A *ZEB2* mRNA m**^**6**^**A with m**^**6**^**A *via* the dm**^**6**^**A CRISPR system inhibited renal tubular cell EMT.***A*, the threshold cycle (Ct) of qPCR showing SELECT results for detecting the m^6^A site in the potential m^6^A site of the *ZEB2* mRNA CDS in sh-control or sh-*METTL3* cells. *B*, the threshold cycle (Ct) of qPCR showing SELECT results for detecting the m^6^A site in the CDS of *ZEB2* mRNA in HK-2 cells transfected with dCas13b-ALKBH5 combined with nontargeting gRNA (NT-gRNA) or gRNA1/2/3 for 24 h. *C*, m^6^A RIP-qPCR analysis of *ZEB2* mRNA in HK-2 cells transfected with gRNAs and dCas13b-ALKBH5. *D*, the mRNA level of *ZEB2* in HK-2 cells transfected with dCas13b-ALKBH5 combined with NT-gRNA or gRNA1/2/3 for 48 h was measured by qRT-PCR. *E*, the protein expression of ZEB2 in HK-2 cells transfected with dCas13b-ALKBH5 combined with NT-gRNA or gRNA1/2/3 for 48 h was measured by Western blot analysis (*left*) and quantified (*right*). *F*, RIP-qPCR analysis of *ZEB2* mRNA in HK-2 cells transfected with dCas13b-ALKBH5 combined with a gRNA control (dC-A5) or gRNA for ZEB2 (dC-A5 + gRNA) for 24 h with antibodies against IGF2BP3. *G*, HK-2 cells were transfected with NT-gRNA, gRNA1 for ZEB2, or dCas13b-ALKBH5 for 24 h and then further treated with Act-D for the indicated times. The mRNA level of *ZEB2* was determined by qRT-PCR. *H*, RIP-qPCR analysis of *ZEB2* mRNA in HK-2 cells transfected with dCas13b-ALKBH5 combined with gRNA control (dC-A5) or gRNA for ZEB2 (dC-A5 + gRNA) for 24 h by use of antibodies against YTHDF1. *I*, HK-2 cells were transfected with vector, a YTHDF1 construct, dCas13b-ALKBH5 combined with NT-gRNA, or dCas13b-ALKBH5 combined with gRNA1 for 48 h, after which the expression of ZEB2 was assessed by Western blot analysis (*left*) and quantitative analysis (*right*). *J*, the wound healing of HK-2 cells transfected with dCas13b-ALKBH5 combined with NT-gRNA or gRNA1 for 48 h was recorded (*left*), and the results were quantitatively analyzed (*right*). (The scale bar represents 200 μm). *K*, the mRNA levels of *α-SMA* and *N-Cad* in HK-2 cells transfected with dCas13b-ALKBH5 combined with NT-gRNA or gRNA1 for 48 h were measured by qRT-PCR. *L*, the protein expression of α-SMA and N-Cad in HK-2 cells transfected with dCas13b-ALKBH5 combined with NT-gRNA or gRNA1 for 48 h was measured by Western blot analysis (*left*) and quantified (*right*). The data are presented as the means ± SDs from three independent experiments. ∗*p <* 0.05, ∗∗*p <* 0.01, ∗∗∗*p <* 0.001, NS, not significant; and Student’s *t* test. α-SMA, α-smooth muscle actin; Act-D, actinomycin-D; ALKBH5, AlkB homolog 5; CDS, coding sequence; EMT, epithelial-to-mesenchymal phenotypic transition; gRNA, guide RNA; gRNA, guide RNA; m^6^A, N6-methyladenosine; METTL3, methyltransferase-like 3; RIP, RNA binding protein immunoprecipitation; ZEB2, zinc finger E-box–binding homeobox 2.

To investigate the role of m^6^A in *ZEB2* mRNA, the SELECT method (45) was used to confirm its modification at A2137. The results showed that A2137 of *ZEB2* mRNA was modified by m^6^A, and its methylation levels were reduced in sh-*METTL3* renal tubular cells (Fig. 6*A*). To further target the demethylation of *ZEB2* mRNA by A2137, we used dm^6^ACRISPR, a fusion of the catalytically dead type VI-B Cas13 enzyme with the m^6^A demethylase ALKBH5, which was developed in our previous study (46). Three guide RNAs (gRNAs) were designed to target *ZEB2* mRNA at distinct positions around the m^6^A site (Fig. S7*A*). The efficiency of the gRNAs was tested using WT Cas13b, which cleaves targeted mRNA (Fig. S7*B*). However, the mRNA levels of ZEB2 in cells transfected with gRNAs alone (Fig. S7*C*) or gRNAs combined with dCas13b (Fig. S7*D*) did not significantly change. The results showed that all three gRNAs effectively bound to the *ZEB2 mRNA*. SELECT-qPCR results indicated that the three gRNAs combined with dCas13b-ALKBH5 reduced the m^6^A levels of *ZEB2* mRNA, with gRNA1 having the strongest effect (Fig. 6*B*). m^6^A-RIP-PCR results also showed that dm^6^ACRISPR induced ZEB2 demethylation (Fig. 6*C*), indicating that the m^6^A at A2137 on *ZEB2* mRNA was significantly removed by the dm^6^ACRISPR system.

Moreover, targeting ZEB2 with a dm^6^ACRISPR resulted in significant downregulation of *ZEB2* mRNA (Fig. 6*D*) and protein (Fig. 6*E*) expression in HK-2 cells. RIP-PCR analysis revealed that the addition of the dm^6^ACRISPR gene to the gRNA for ZEB2 could significantly decrease the binding of *ZEB2* mRNA to IGF2BP3 (Fig. 6*F*) and destabilize its mRNA (Fig. 6*G*). Additionally, the addition of the dm^6^ACRISPR gene to the gRNA for ZEB2 significantly decreased the binding of *ZEB2* mRNA to YTHDF1 (Fig. 6*H*), and the overexpression of YTHDF1 had no further effect on ZEB2 protein expression in HK-2 cells treated with the dm^6^ACRISPR sequence with the gRNA (Fig. 6*I*).

We further investigated the potential effect of the dm^6^ACRISPR-mediated targeting of ZEB2 to m^6^A on the EMT of renal tubular cells. Our data showed that the dm^6^ACRISPR targeting m^6^Aof ZEB2 m^6^A can suppress the migration (Fig. 6*J*) of HK-2 cells. Consistently, it also downregulated the mesenchymal markers α-SMA and N-Cad in HK-2 cells (Fig. 6, *K* and *L*). These data confirmed that targeting the m^6^A modification of ZEB2 *via* the dm^6^ACRISPR system can suppress ZEB2 expression and impair the EMT of renal tubular cells.

### m^6^A regulates *in vivo* RF and is associated with RF clinical progression

**Figure 7:**
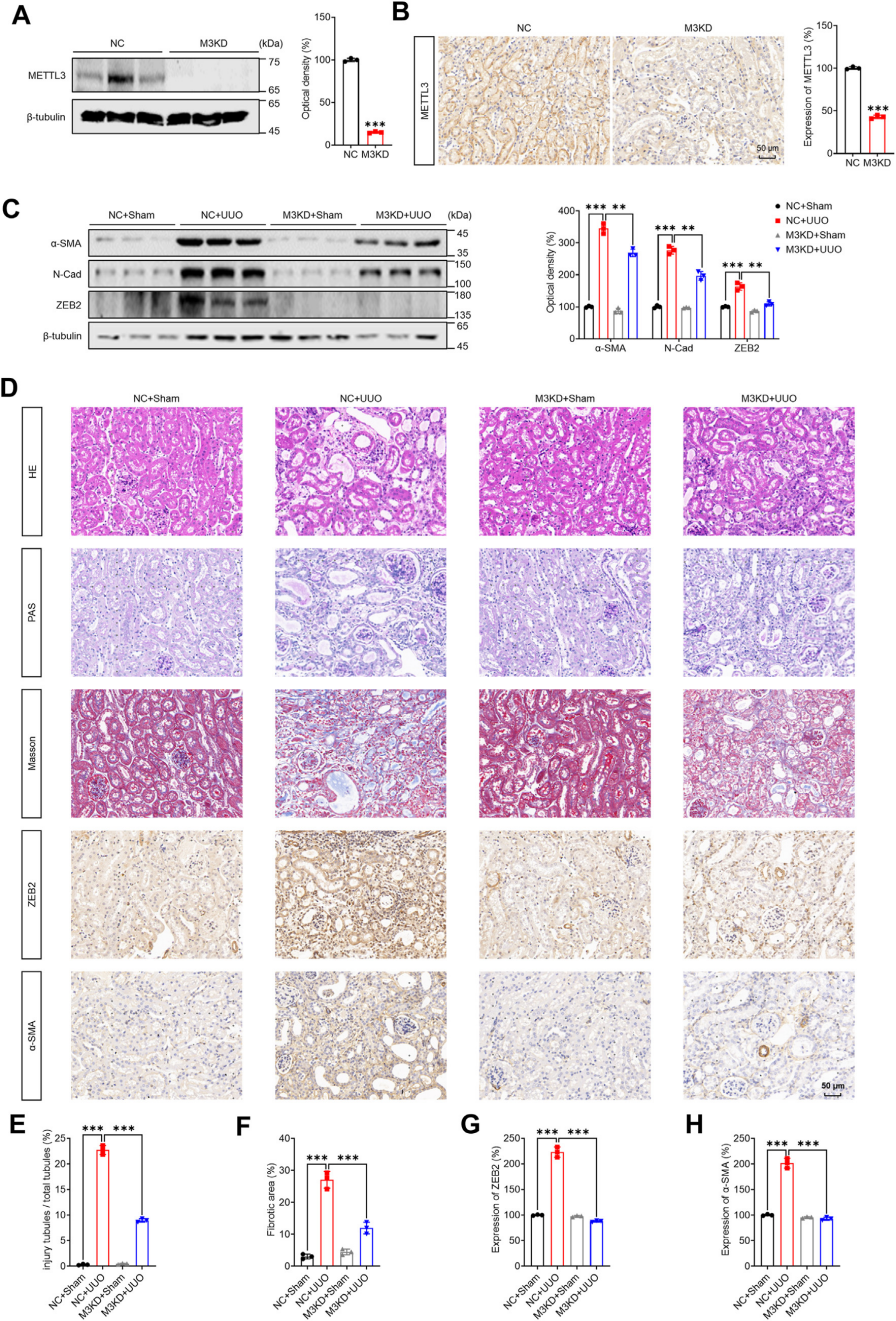
**METTL3/ZEB2 axis regulated *in vivo* RF development.***A*, Western blot analysis of METTL3 expression in METTL3-knockdown mice compared with control mice. *B*, immunohistochemistry analysis of METTL3 expression in METTL3-knockdown mice compared with control mice. (The scale bar represents 50 μm.) *C*, the protein levels of α-SMA, N-Cad, and ZEB2 in the UUO 14-day model with or without METTL3 knockdown were measured by Western blot analysis (*left*) and quantified (*right*). *D*–*H*, H&E, PAS, Masson’s trichrome, and immunohistochemistry staining of UUO model mice with or without METTL3 knockdown at 14 days (*D*) and quantitative analysis *E–H*). (The scale bar represents 50 μm.) The data are presented as the means ± SDs from three independent experiments. ∗*p <* 0.05, ∗∗*p <* 0.01, ∗∗∗*p <* 0.001, NS, not significant; and Student’s *t* test. α-SMA, α-smooth muscle actin; METTL3, methyltransferase-like 3; PAS, periodic acid-Schiff; RF, renal fibrosis; UUO, unilateral ureteral obstruction; ZEB2, zinc finger E-box–binding homeobox.

To investigate the role of m^6^A in RF *in vivo*, we utilized an AAV9-packaged METTL3 knockdown plasmid to silence METTL3 in mice. The depletion of METTL3 in TECs was verified through both Western blot analysis (Fig. 7*A*) and immunofluorescence (Fig. 7*B*). The protein levels of N-Cad, α-SMA, and ZEB2 were increased in the 14-day unilateral ureteral obstruction (UUO 14 days) model and were decreased by the deletion of METTL3 (Fig. 7*C*). Additionally, H&E, Masson, periodic acid-Schiff, and immunohistochemical staining revealed the loss of normal interstitial structure, dilated renal tubules, swollen renal tubular cells, varying degrees of degeneration, and even necrosis, as well as significantly increased interstitial collagen fibril deposition in the UUO 14-day model. However, the deletion of METTL3 significantly reduced renal tubular damage and interstitial collagen fibril deposition in the tubules (Fig. 7, *D*–*H*). These findings were further confirmed in the unilateral ischemia-reperfusion injury model (Fig. S8, *A*–*F*). The deletion of METTL3 also reduced unilateral renal ischemia reperfusion injury (UIRI)-induced RF while decreasing serum blood urea nitrogen and serum creatinine concentrations (Fig. S8, *G* and *H*). Collectively, these data suggested that m^6^A positively regulates RF *in vivo*.

**Figure 8:**
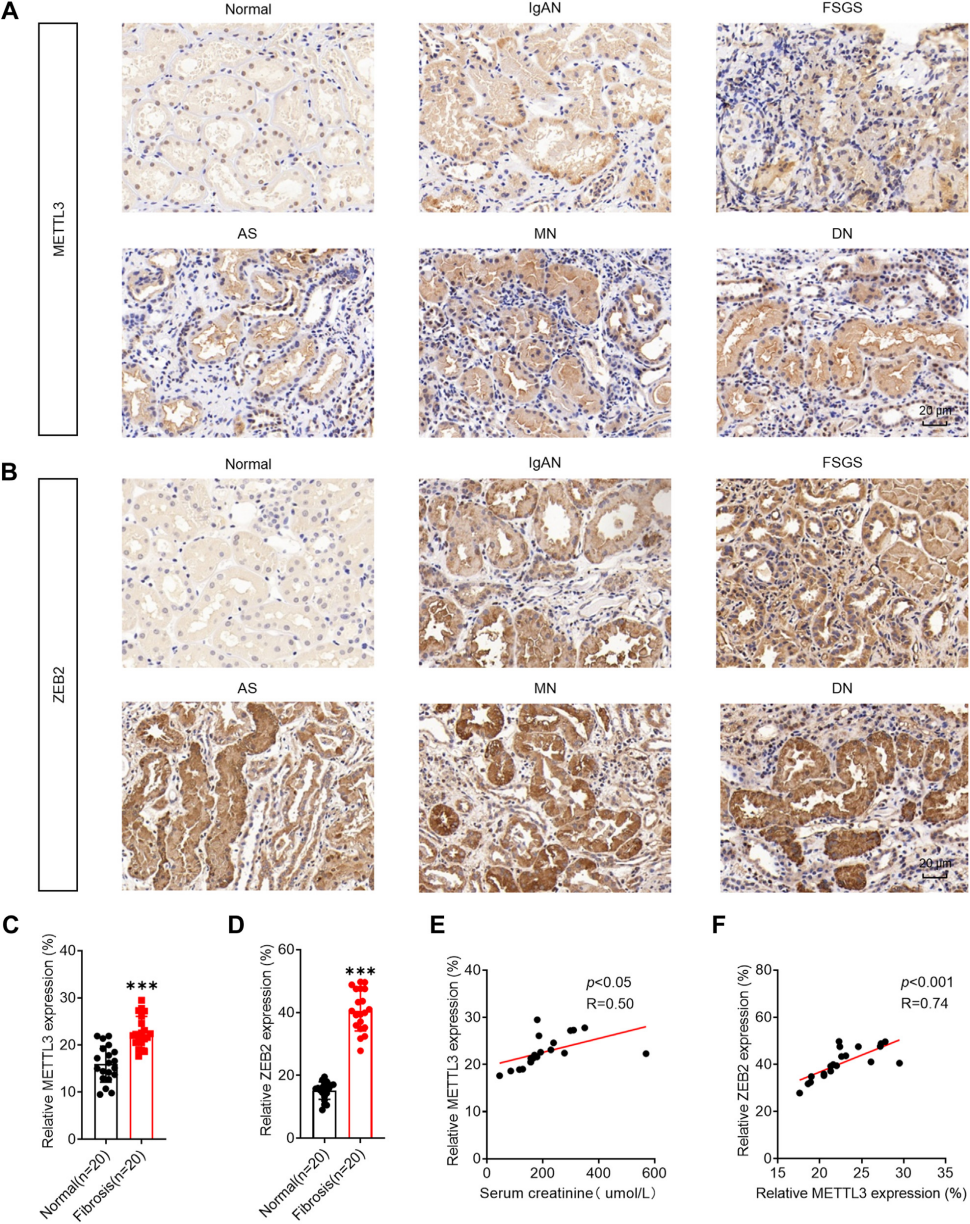
**The METTL3/ZEB2 axis was associated with RF clinical progression.***A*, representative immunohistochemistry staining images of METTL3 in kidney biopsies from patients with RF. (The scale bar represents 20 μm). *B*, representative immunohistochemistry staining data of ZEB2 in kidney biopsies from patients with RF. (The scale bar represents 20 μm). *C*, correlation of METTL3 expression between patients with RF and renal carcinoma-adjacent normal kidney tissues. *D*, the correlation between the expression of ZEB2 in patients with RF and that in renal carcinoma-adjacent normal kidney tissues. *E*, correlation between Scr and METTL3 protein levels in patients with RF. *F*, correlation between METTL3 and ZEB2 protein expression in patients with RF. The data are presented as the means ± SDs from three independent experiments. ∗*p <* 0.05, ∗∗*p <* 0.01, ∗∗∗*p <* 0.001, NS, not significant; and Student’s *t* test. METTL3, methyltransferase-like 3; RF, renal fibrosis; Scr, serum creatinine; ZEB2, zinc finger E-box–binding homeobox 2.

To further investigate the clinical relevance of METTL3 in the pathogenesis of RF, we performed immunostaining on kidney biopsy specimens from 20 patients with RF due to various etiologies, including focal segmental glomerulosclerosis, IgA nephropathy, diabetic nephropathy, and membranous nephropathy (Fig. 8, *A* and *B*). The clinical data showed that the expression of METTL3 (Fig. 8*C*) and ZEB2 (Fig. 8*D*) in RFs was significantly greater than that in adjacent normal tissues from renal cell carcinoma patients. A significant increase in the expression level of METTL3 was observed in RF biopsy patients with an increase in creatinine (Fig. 8*E*). Moreover, immunohistochemical analysis confirmed a positive correlation between METTL3 and ZEB2 expression in 20 RF patients (Fig. 8*F*). These results further supported the notion that m^6^A is positively associated with the progression of RF.

## Discussion

The EMT of renal tubular cells is generally thought to promote RF progression (47, 48). Our present study showed that m^6^A positively regulated RF by promoting EMT in renal tubular cells, while knockdown of METTL3 suppressed the progression of RF. Consistently, recent studies have indicated that UUO-induced fibrosis in mouse kidneys results in the adverse expression of RF-related proteins and significant increases in the total m^6^A level, while genistein ameliorates RF through the regulation of Snail *via* the m^6^A RNA demethylase ALKBH5 (49). Furthermore, inhibition of METTL3 attenuates renal injury and inflammation by alleviating TAB3 m^6^A modifications *via* IGF2BP2-dependent mechanisms (26). In addition, METTL3 was found to play a major catalytic role in m^6^A modification in UUO mice and to drive obstructive RF development by promoting miR-21-5p maturation (50). Our present data, together with published results, confirmed that m^6^A and METTL3 have positive effects on RF progression. These findings also support the possibility of developing therapeutic strategies against RF progression by targeting m^6^A modifications.

We found that m^6^A triggers the EMT of renal tubular cells by increasing the mRNA stability and translation of *ZEB2* mRNA. ZEB2 is an important transcription factor during the EMT process that binds to the E-Cad promoter and represses its expression, thereby promoting EMT (51). ZEB2 plays a role in the TGF-β signaling pathway through Smad proteins (51). Recent studies have indicated that ZEB2 is upregulated in hypoxic podocytes and in the pathogenesis of fibrotic diseases involving collagen, such as Mowat–Wilson syndrome and embryogenesis of renal tubules (52, 53). Taken together, these data suggested that the expression of ZEB2 during RF is regulated by m^6^A, and targeted inhibition of the METTL3/ZEB2 pathway might be a potential approach for suppressing RF progression.

Regarding the mechanism responsible for the m^6^A-mediated regulation of ZEB2 expression, we found that m^6^A increased ZEB2 expression by positively regulating ZEB2 translational elongation and mRNA stability by binding to the YTHDF1/eEF-2 complex and IGF2BP3, respectively. m^6^A can regulate pre-mRNA splicing and nuclear export and affect mRNA stability and translation (54, 55, 56). Previous studies have also indicated that m^6^A methylation at specific mRNAs may have multiple functions. For example, m^6^A at the Snail CDS can decrease mRNA stability while enhancing translation efficiency (57). In addition, IGF2BP-regulated m^6^A read can promote both mRNA stability and translation (42). Our study revealed that m^6^A enhances both the mRNA stability and translation efficiency of *ZEB2* mRNA, which synergistically promotes the expression of ZEB2 in renal tubular cells.

We further provided *in vivo* evidence and clinical data to support the role of m^6^A/ZEB2 in promoting renal tubular cell EMT and RF progression. Intriguingly, targeted demethylation of *ZEB2* mRNA by the dm^6^ACRISPR system can suppress the expression of ZEB2 and inhibit EMT progression in renal tubular cells. These findings indicated that specific targeted genes could change the phenotype of human cells, which has great potential for treating human diseases, including RF. Similarly, previous studies indicated that targeted demethylation of PDK4 suppresses glycolysis (42), while m^6^A sites of HRas proto-oncogene, GTPase targeted by the dm^6^ACRISPR system can decrease cancer proliferation and metastasis (58). All these data suggested that m^6^A/ZEB2 could be potent targets for the development of RF drugs. Furthermore, the targeted demethylation of specific mRNAs could also be a potential approach for RF treatment.

In conclusion, we provided compelling *in vitro* and *in vivo* evidence that m^6^A could regulate EMT in renal tubular cells through the regulation of ZEB2. CDS methylation is essential for m^6^A-mediated mRNA stability and translation. As a large number of genes are involved in EMT, we cannot exclude the possibility that m^6^A modification regulates metabolic processes by indirectly targeting other genes. Our studies showed that m^6^A positively regulates RF through the induction of ZEB2, expanding our understanding of interactions that are critical for therapeutic applications.

## Experimental procedures

### Patients

Renal biopsy specimens were obtained from 20 patients diagnosed with RF due to various causes, along with 20 adjacent normal tissues from patients with renal cell carcinoma, all of which were collected with written informed consent. The clinical parameters of the 40 patients are detailed in Table S1. Immunohistochemistry was performed to evaluate the expression of METTL3 (Abcam, ab195352) and ZEB2 (Abcam, ab124512). The study adhered to the principles of the Declaration of Helsinki and was approved by the Ethics Committee of ZhuJiang Hospital, Southern Medical University (Approval No. 2022-KY-204-02).

### Animal studies

Male C57BL/6 mice, aged 6 to 8 weeks and weighing 22 to 24 g, were obtained from the ZhuJiang Hospital of Southern Medical University Animal Center. The animal procedures adhered to the Guide for the Care and Use of Laboratory Animals and received approval from the Animal Experimentation Ethics Committee of the ZhuJiang Hospital of Southern Medical University Animal Center (Approval No. LAEC-2022-145).

Adeno-associated viruses (AAVs) for AAV9-mediated METTL3 knockdown in mice were developed and obtained from Heyuan. The anesthetized mice received 100 μl of AAV vector containing either the METTL3 gene (pAAV-U6-sh*METTL3*-cytomegalovirus-EGFP-the woodchuck hepatitis post-transcriptional regulatory element, 5 × 10^12^ vg/ml) or the negative control (pAAV-U6-sh-control-cytomegalovirus-EGFP-the woodchuck hepatitis post-transcriptional regulatory element, 5 × 10^12^ vg/ml) *via* injection into the renal pelvis.

To establish the UUO model, male C57BL/6 mice were used. In anesthetized mice, the left ureter was double ligated using 4–0 silk. In the sham-operated mice, the ureters were exposed but not ligated. Fourteen days after UUO, the mice were euthanized. The mice were divided into four groups: (i) mice injected with negative control AAVs and used as the sham-operated model, (ii) mice injected with AAV vectors carrying the METTL3 gene and used as the sham-operated model, (iii) mice injected with negative control AAVs and used as the UUO 14-day model, and (iv) mice injected with AAV vectors carrying the METTL3 gene and used as the UUO 14-day model.

For the UIRI model, male C57BL/6 mice were used. In anesthetized mice, the left renal ureter was clipped with microaneurysm clamps for 35 min. The clamps were then removed to allow reperfusion of the kidneys. Ten days later, the right kidney was removed. Sham-operated mice underwent perirenal capsule dissection but not nephrectomy. Mice were euthanized 11 days after UIRI. The mice were divided into four groups: (i) mice injected with negative control AAVs and used as a sham-operated model, (ii) mice injected with an AAV vector carrying the METTL3 gene and used as a sham-operated model, (iii) mice injected with negative control AAVs and used as a UIRI model, and (iv) mice injected with an AAV vector carrying the METTL3 gene and used as a UIRI model.

Postsurgery, blood samples were collected to analyze serum creatinine and blood urea nitrogen levels. Kidneys were either preserved in liquid nitrogen or fixed overnight in 4% paraformaldehyde, followed by embedding in paraffin. Histological evaluation included H&E staining for detecting pathological changes, Masson's trichrome staining for assessing RF, and periodic acid-Schiff staining for visualizing renal tubular injury. Immunohistochemical analysis was conducted using specific antibodies against METTL3 (Abcam, ab195352), ZEB2 (Abcam, ab124512), and α-SMA (Abcam, ab5694), and the sections were examined under a microscope.

### Cell culture, treatments, and transfection

The human proximal TEC lines HK-2 and HKC-8, which were kindly provided by Prof. Lili Zhou at NanFang Hospital of Southern Medical University, were cultured in Dulbecco's modified Eagle's medium (Gibco, Thermo Fisher Scientific, Inc) supplemented with 10% fetal bovine serum (Gibco) and 1% penicillin/streptomycin (Invitrogen) at 37 °C with 5% CO_2_. To establish a model of renal tubular cells undergoing EMT, the cells were treated with 10 ng/ml TGF-β for 3 days. To inhibit METTL3 activity, cells were treated with 5 ng/ml of the METTL3 inhibitor STM2457. Stable cell lines, including sh-control and sh-*METTL3* HK-2 cells and sh-control and sh-*METTL3* HKC-8 cells, were generated by virus packaging. Plasmids were transfected into cells using Lipofectamine 3000 reagent (Invitrogen) following the manufacturer's instructions for the overexpression of ALKBH5, ZEB2, YTHDF1, or IGF2BP3. The knockdown of YTHDF1, METTL3, or IGF2BP3 was achieved by transfection with siRNA using Lipofectamine 3000 reagent (Invitrogen) according to the manufacturer's instructions. The sequences of the siRNAs used are listed in Table S2.

### RNA-sequencing

Total RNA was isolated from sh-control or sh-*METTL3* HK-2 cells treated with TRIzol reagent using an RNeasy Mini Kit (Qiagen). mRNA preparation and sequencing were performed by Novogene. TopHat (version 2.0.6) was used to map all sequencing reads to the reference human genome sequence (NCBI36.1 [hg19] assembly).

### Wound healing

The cells were seeded and cultured until they formed a 90% confluent monolayer. Subsequently, a sterile pipette tip was used to scratch the cells, and the cells were cultured in medium lacking fetal bovine serum. The distances migrated by the cells into the scratched area were photographed and recorded at the specified time points, as per the experimental design.

### LC-MS/MS assay

The mRNA was isolated using oligo dT magnetic beads. Subsequently, nuclease P1 (0.5 U, Sigma) was added to a 25 μl reaction mixture containing 10 mM NH_4_OAc (pH = 5.3) and incubated at 42 °C for 1 h to digest the mRNA. The digested mRNA was then mixed with NH_4_HCO3 (1 M, 3 μl) and alkaline phosphatase (1 μl, 1 U/μl; Sigma) and incubated at 37 °C for 1 h. The samples were separated on a C18 column (Agilent) using an Agilent 6410 QQQ triple quadrupole liquid chromatography mass spectrometer. The m^6^A to A ratio was calculated based on calibration curves.

### Dot-blot assay

mRNA extraction was carried out using oligo dT magnetic beads, which were subjected to denaturation at 95 °C for a period of 5 min. Next, the mRNA was immobilized onto a nylon membrane through ultraviolet cross-linking. Next, the membrane was blocked using 5% nonfat milk and then probed overnight with an m^6^A antibody (Synaptic Systems, 202003) at 4 °C. After standard washing, the membrane was incubated with secondary antibody at room temperature for 1 h. Finally, the signals were detected using the Gel Imaging Analysis System 5200.

### N6-methyladenosine-RIP-PCR

Protein G magnetic beads were incubated with 1 μg of m^6^A or IgG antibody in 1 × reaction buffer (150 mM NaCl, 10 mM Tris–HCl, pH 7.5, 0.1% NP-40 in nuclease-free H2O) at 4 °C for 3 h. Next, the conjugated beads were incubated with 200 μg of extracted RNA at 4 °C for 3 h. To elute the bound RNAs, the RNA-antibody–conjugated beads were incubated with 100 μl of elution buffer (75 mM NaCl, 50 mM Tris–HCl, pH 7.5, 6.25 mM EDTA, 1% (w/v) SDS, 20 mg/ml proteinase K) for 30 min at room temperature. The eluted RNA was extracted using the phenol: chloroform method and ethanol precipitation. The isolated m^6^A-RIP RNA was reverse-transcribed and quantified by qPCR. The immunoprecipitation enrichment ratio of a transcript was calculated as the ratio of its amount in the IP to that in the input obtained from the same number of cells.

### RIP-PCR

Prior to the harvesting process, the cells were exposed to UV cross-linking for half an hour. Next, a lysate containing 200 mg of total protein, along with protease and RNase inhibitors, was incubated with either the antibody or the IgG-conjugated Dynabeads Protein G (Thermo Fisher Scientific) for a period of 3 h. The RNA that was pulled down by the antibody was subsequently eluted and dissolved in TRIzol (Invitrogen) and then subjected to ethanol precipitation. The concentration of RNA was determined using the Qubit RNA HS Assay Kit (Thermo Fisher Scientific). For quantitative real-time PCR analysis, 2 ng of total RNA and immunoprecipitation RNA were utilized as templates.

### RNA and protein stability

Posttranscriptional control of gene expression is critical in a number of cells. The first stage of RNA splicing is the process of removing introns from pre-mRNA and linking exons to produce mature mRNA. Act-D was introduced to limit RNA synthesis during cell culture, hence blocking the RNA transcription process. Pre-RNA cannot be produced or cleaved into mRNA. After various durations of action, the total RNA of cells can be collected, and the expression levels of mRNA and precursor RNA can be dynamically identified using real-time fluorescence quantitative technology, allowing for the analysis of mRNA and precursor RNA expression changes.

sh-control or sh-*METTL3* HK-2 cells were seeded 1 day prior to treatment. For the RNA stability assay, Act-D was added to the cell culture medium at a concentration of 5 mg/ml, and the cells were treated for specific time periods. Total RNA was extracted from the cells and quantified using quantitative real-time PCR to assess RNA stability. For the protein stability assay, cells were treated with CHX at a concentration of 100 mg/ml for specific time periods. The cells were then lysed in 1X SDS loading solution, and the protein stability was analyzed *via* Western blotting.

### Luciferase reporter assay

The CDS sequence was subcloned into the pmiGLO dual-luciferase vector. Mutagenesis of m^6^A sites (A–G) was performed with a site-directed mutagenesis kit (Thermo Fisher Scientific). F-Luc activity measurements were standardized to renilla luciferase (R-luc) activity values to represent expression efficiency. The translation outcome was assessed by comparing the F-luc/R-luc signal to mRNA abundance, and translation efficiency was defined as the ratio of reporter protein production to mRNA abundance. A luciferase assay was performed using reported lysis buffer (Catalog #E3971, Promega) according to the manufacturer's instructions. sh-control or sh-*METTL3* HK-2 cells were transfected with pmirGLO, pmirGLO-WT-CDS, pmirGLO-Mut1-CDS, or pmirGLO-Mut2-CDS in a 6-well plate. After 6 h of transfection, the cells were transferred to a 96-well plate and incubated for 24 h. The Dual-Glo Luciferase Assay System (Promega) was used to measure luciferase activity. We normalized F-luc activity to R-luc activity to evaluate reporter translation efficiency.

### Polysome profiling

Cells were treated with 100 mg/ml CHX for 2 min at 37 °C before being harvested. Following a wash with PBS-CHX, the cells were lysed using 300 ml of lysis buffer composed of 140 mM NaCl, 5 mM MgCl2, 10 mM Tris–HCl (pH 8.0), 1% Triton X-100, 0.5% sodium deoxycholate, 0.4 U/ml RNase inhibitor, 20 mM DTT, 0.1 mg/ml CHX, 10 mM ribonucleoside vanadyl complexes, and 0.1% cocktail. The resulting cell lysate was obtained through centrifugation at 16,000×*g* for 10 min and then treated with DNase for 10 min at room temperature. Afterward, the DNase-treated cell lysate was applied to a 10 ml 5% to 50% sucrose solution and centrifuged at 170,000×*g* for 2 h at 4 °C using Triax (Biocomp Instruments) to separate ribosomal fractions by gradient profiling. RNA from each polysome fraction was extracted using TRIzol, and its concentration was determined using a Qubit RNA HS Assay Kit (Thermo Fisher Scientific).

### Design of gRNAs

To design gRNAs for targeting the CDS region of ZEB2, the mRNA sequences of all isoforms of the target genes were analyzed to identify common regions. MEGABLAST (https://blast.ncbi.nlm.nih.gov/Blast.cgi) was used to check all the designed gRNAs to avoid mismatches with unexpected mRNAs in the human genome. The sequences of the three selected gRNAs were as follows: gRNA1, 5′-AAGGAGTATTACTCCTGGAGTGGTCCAATTTTTCAACTGG-3′; gRNA2, 5′-AGAAACACTGTTATGATCTAAACTGATGCTACTAGCTTTT-3′; and gRNA3, 5′-ATCTTTTTGCGAGACAGACAGGAGTCGGAGTCTGTCATAT-3′.

### SELECT qPCR

The SELECT qPCR method was performed according to Xiao's protocol (29) with some modifications，which is a single-base elongation- and ligation-based qPCR amplification method that exploits the ability of m^6^A to hinder 1) the single-base elongation activity of DNA polymerases and 2) the nick ligation efficiency of ligases.

Total RNAs were quantified using a Qubit (Thermo Fisher Scientific) and QubitTM RNA HS Assay Kit (Thermo Fisher Scientific). Next, in 17 μl of 1 × CutSmart buffer (NEB), 1500 ng of total RNA, 40 nM up and down primers, and 5 μM deoxy-ribonucleoside triphosphate were combined. The following schedule for incubating the combination was used: 90 °C for 1 min, 80 °C for 1 min, 70 °C for 1 min, 60 °C for 1 min, 50 °C for 1 min, and 40 °C for 6 min. In addition, the sample was combined with 0.5 U SplintR ligase, 10 nM ATP, and 3 μl of 0.01 U Bst 2.0 DNA polymerase. It was then incubated for 20 min at 40 °C and denatured for 20 min at 80 °C. Following that, a 20 μl qPCR reaction was carried out using 2 μl of the final reaction mixture, 2 × SYBR Green Master Mix (TaKaRa), and 200 nM SELECT primers (found in Table S3). 95 °C, 5 min; (95 °C, 10 s; 60 °C, 35 s) × 40 cycles; 95 °C, 15 s; 60 °C, 1 min; 95 °C, 15 s; and 4 °C hold were the parameters for the qPCR procedure. The Ct values of the samples were standardized to the corresponding Ct values of the control to determine the results. Three separate experiments were used for each assay.

### Western blot analysis

Total cell lysates were collected as described previously (57). The primary antibodies used for immunoblotting included anti-α-SMA (Proteintech, 14395-1-AP), anti-N-Cad (Proteintech, 22018-1-AP), anti-METTL3 (Abcam, ab221795), anti-METTL3 (Proteintech, 15073-1-AP), anti-METTL14 (Abcam, ab220030), anti-ALKBH5 (Abcam, ab234528), anti-FTO (Abcam, ab92821), anti-ZEB2 (Abcam, ab191364), anti-ZEB2 (Proteintech, 14026-1-AP), anti-YTHDF1 (Proteintech, 17479-1-AP), anti-YTHDF2 (Proteintech, 24744-1-AP), anti-YTHDF3 (Proteintech, 25537-1-AP), anti-IGF2BP1 (Proteintech, 22803-1-AP), anti-IGF2BP2 (Proteintech, 11601-1-AP), anti-IGF2BP3 (Proteintech, 14642-1-AP), anti-eEF1 (Proteintech, 11402-1-AP), anti-eEF2 (Proteintech, 20107-1-AP), anti-m^6^A (Cell Signaling Technology, D9D9W), anti-H2AX (Proteintech, 10856-1-AP), anti-GAPDH (Proteintech, 10494-1-AP), and anti-β-tubulin (Proteintech, 10094-1-AP). The presented immunoblot results are representative of at least three independent experiments.

### RT-PCR and real-time PCR

RNA extraction was performed using TRIzol (Invitrogen), and quantitative real-time PCR was conducted as previously described (46). GAPDH was used as a control for normalization. In the nuclear fraction and Act-D–treated samples, hypoxanthine phosphoribosyl transferase and 18S served as controls for normalization. The primers used for quantitative real-time PCR are listed in Table S4.

### Co-immunoprecipitation assay

The cells were pelleted and lysed in 400 μl of radio immunoprecipitation assay lysis buffer supplemented with protease inhibitors. The clear lysate was precleaned using 20 ml of Dynabeads protein G (Thermo Fisher Scientific) for 2 h at 4 °C. The precleaned cell lysate was then incubated with antibody- or IgG-conjugated Dynabeads protein G at 4 °C overnight. The beads were rinsed three times with radio immunoprecipitation assay lysis buffer and boiled in 30 ml of 1× SDS loading dye. The eluted samples were analyzed by Western blot.

### Statistical analyses

The data are reported as the mean ± SD from at least three independent experiments. Statistical analysis was performed using a two-tailed unpaired Student’s *t* test for comparisons between two groups and one-way or two-way ANOVA followed by the Bonferroni correction for multiple comparisons. All the statistical tests were two-sided. The data analysis was conducted using SPSS 16.0 for Windows, and a *p* value of <0.05 was considered to indicate statistical significance (∗*p* < 0.05, ∗∗*p* < 0.01, ∗∗∗*p* < 0.001; and NS, no significance).

## Conclusion

This study suggested that m^6^A triggers the progression of RF *via* ZEB2, which provides a new therapeutic target for RF treatment while expanding our understanding of the role of mRNA methylation in kidney disease.

## Data availability

The datasets used and analyzed during the current study are available from the corresponding author upon reasonable request.

## Supporting information

This article contains supporting information.

## Conflict of interest

The authors declare that they have no conflicts of interest with the contents of this article.
