# Supplementary material for: N6-methyladenosine triggers renal fibrosis via enhancing translation and stability of ZEB2 mRNA
Source: J Biol Chem. 2024 Jul 24;300(9):107598. doi: 10.1016/j.jbc.2024.107598 (PMC11381876; doi:10.1016/j.jbc.2024.107598)
Supplement: Supplementary Materials [file mmc6.docx]

**
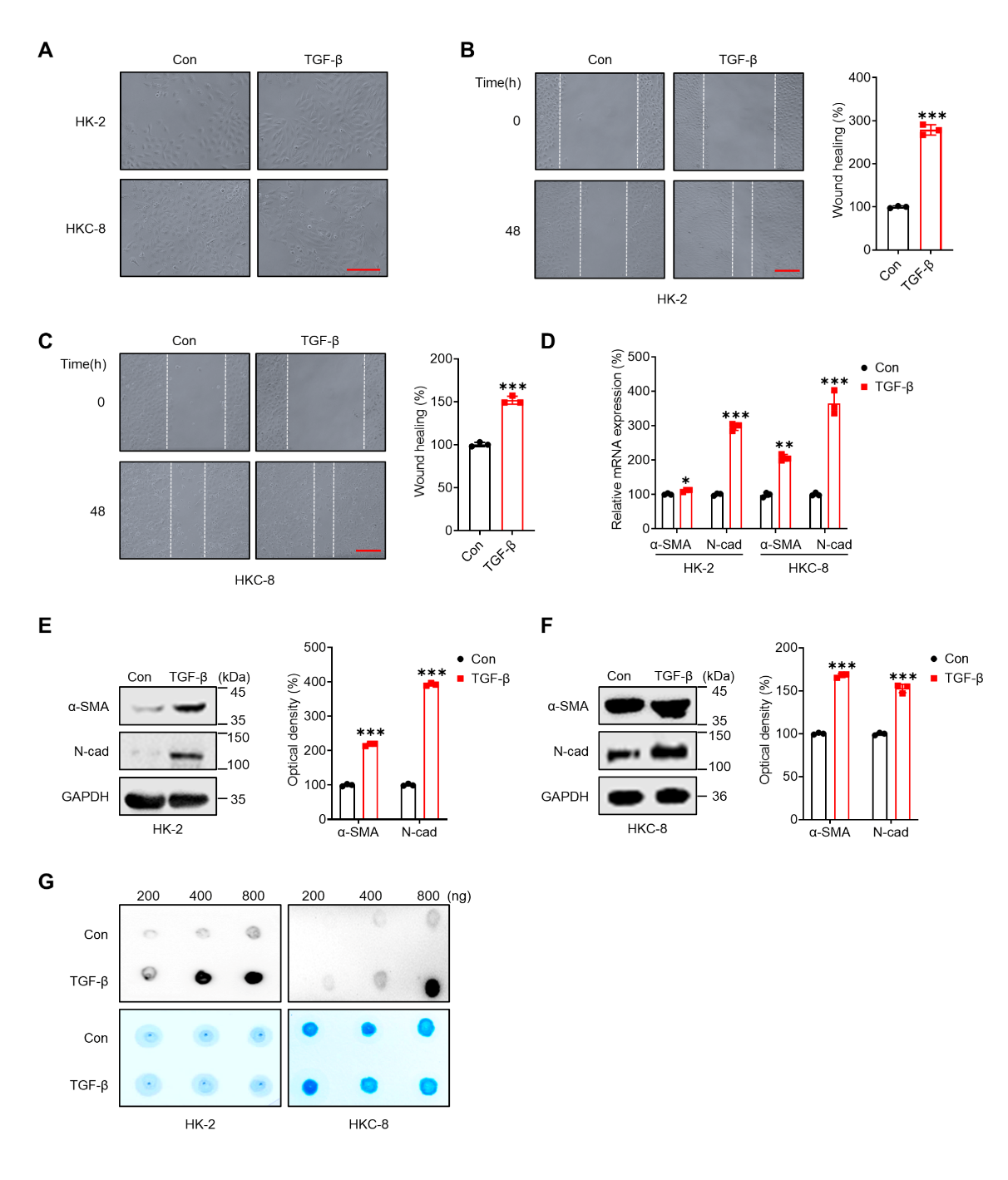
**

**Supplementary Figure1. m6A was involved in EMT of renal tubular cells**

1. HK-2 or HKC-8 cells were treated with or without 10 ng/ml TGF-β for 3 days, the phenotypic changes of cells were recorded by phase contrast microscope.
2. HK-2 cells were treated with or without 10 ng/ml TGF-β for 48 h, wound healing was recorded (left) and quantitatively analyzed (right).
3. HKC-8 cells were treated with or without 10 ng/ml TGF-β for 48 h, wound healing was recorded (left) and quantitatively analyzed (right).
4. HK-2 or HKC-8 cells treated with or without 10 ng/ml TGF-β for 24h, the mRNA of α-SMA and N-Cad were measured by qRT-PCR.
5. HK-2 cells treated with or without 10 ng/ml TGF-β for 48h, the protein levels of α-SMA and N-Cad were measured by western blot analysis (left) and quantitatively analyzed (right).
6. HK-2 cells treated with or without 10 ng/ml TGF-β for 48h, the protein levels of α-SMA and N-Cad were measured by western blot analysis (left) and quantitatively analyzed (right).
7. Dot-blot analysis of m6A levels in total mRNA of HK-2 or HKC-8 cells treated with or without 10 ng/ml TGF-β for 3 days.

Data are presented as means ± SD from three independent experiments. *p < 0.05, **p < 0.01, ***p < 0.001, NS, no significant, by Student’s t test. Red bar = 200 μm


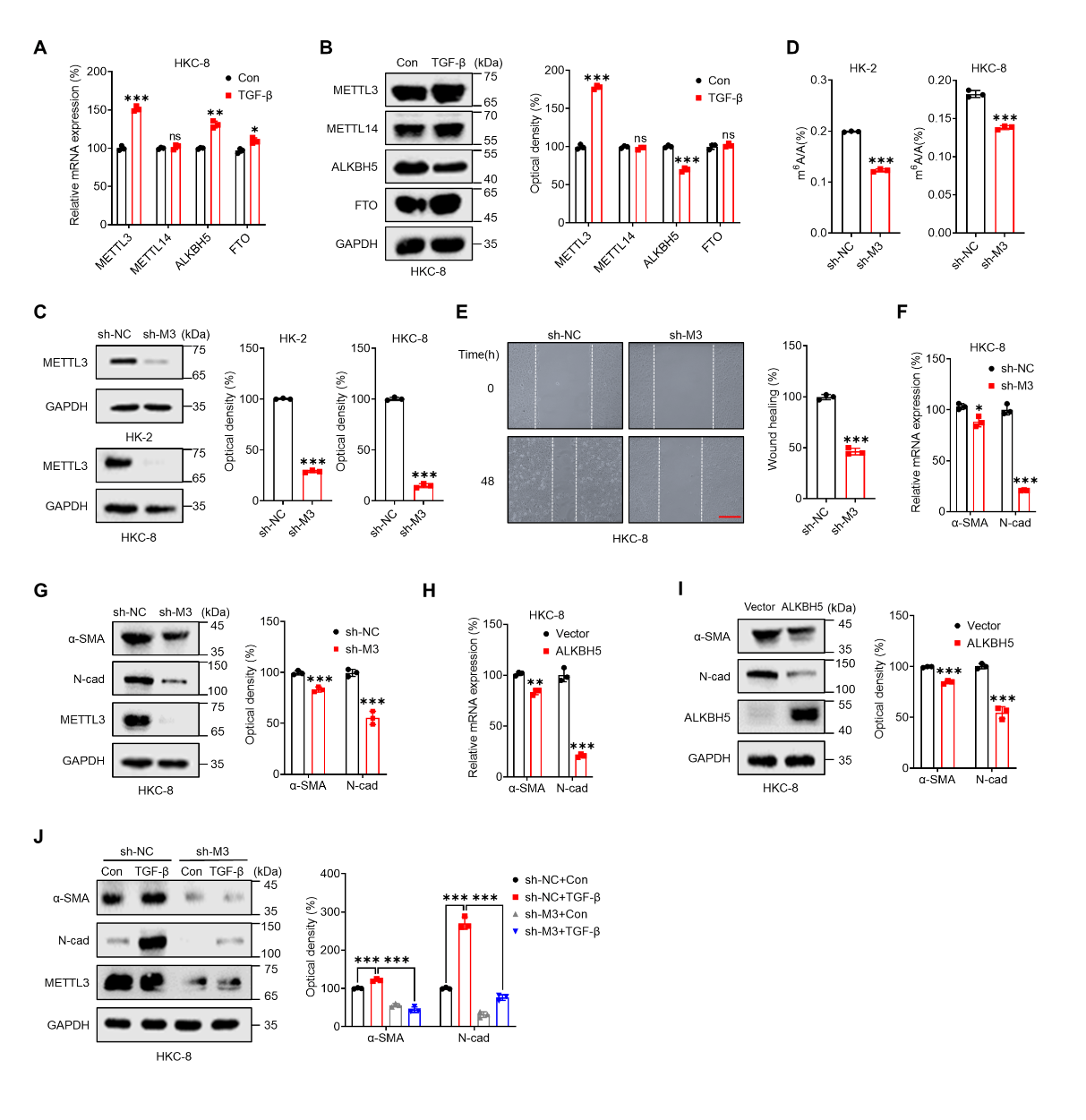


**Supplementary Figure2. Suppression of METTL3 inhibited EMT of renal tubular cells**

1. HKC-8 cells treated with or without 10 ng/ml TGF-β for 24h, the mRNA of m6A methyltransferases (METTL3, METTL14) and m6A demethylases (ALKBH5, FTO) were measured by qRT-PCR.
2. HKC-8 cells treated with or without 10 ng/ml TGF-β for 48h, the protein levels of m6A methyltransferases (METTL3, METTL14) and m6A demethylases (ALKBH5, FTO) were measured by western blot analysis (left) and quantitatively analyzed (right).
3. The protein levels of METTL3 in sh-control (sh-NC) / sh-METTL3 (sh-M3) HK-2 or HKC-8 cells were measured by western blot analysis (left) and quantitatively analyzed (right).
4. The m6A/A ratio of total mRNA in sh-control / sh-METTL3 HK-2 or HKC-8 cells.
5. Wound healing of sh-control or sh-METTL3 HKC-8 cells was recorded (left) and quantitatively analyzed (right).
6. The mRNA of α-SMA and N-Cad in sh-control or sh-METTL3 HKC-8 cells were measured by qRT-PCR.
7. The protein levels of α-SMA and N-Cad in sh-control or sh-METTL3 HKC-8 cells were measured by western blot analysis (left) and quantitatively analyzed (right).
8. HKC-8 cells were transfected with pcDNA (vector) or pcDNA/ALKBH5 for 24 h, the mRNA levels of α-SMA and N-Cad were measured by qRT-PCR.
9. HKC-8 cells were transfected with pcDNA (vector) or pcDNA/ALKBH5 for 48 h, the protein expressions of α-SMA and N-Cad were measured by western blot analysis (left) and quantitatively analyzed (right).
10. sh-control or sh-METTL3 HKC-8 cells were treated with or without 10 ng/ml TGF-β for 3 days, protein levels of α-SMA and N-Cad were measured by western blot analysis (left) and quantitatively analyzed (right).

Data are presented as means ± SD from three independent experiments. *p < 0.05, **p < 0.01, ***p < 0.001, NS, no significant, by Student’s t test. Red bar = 200 μm


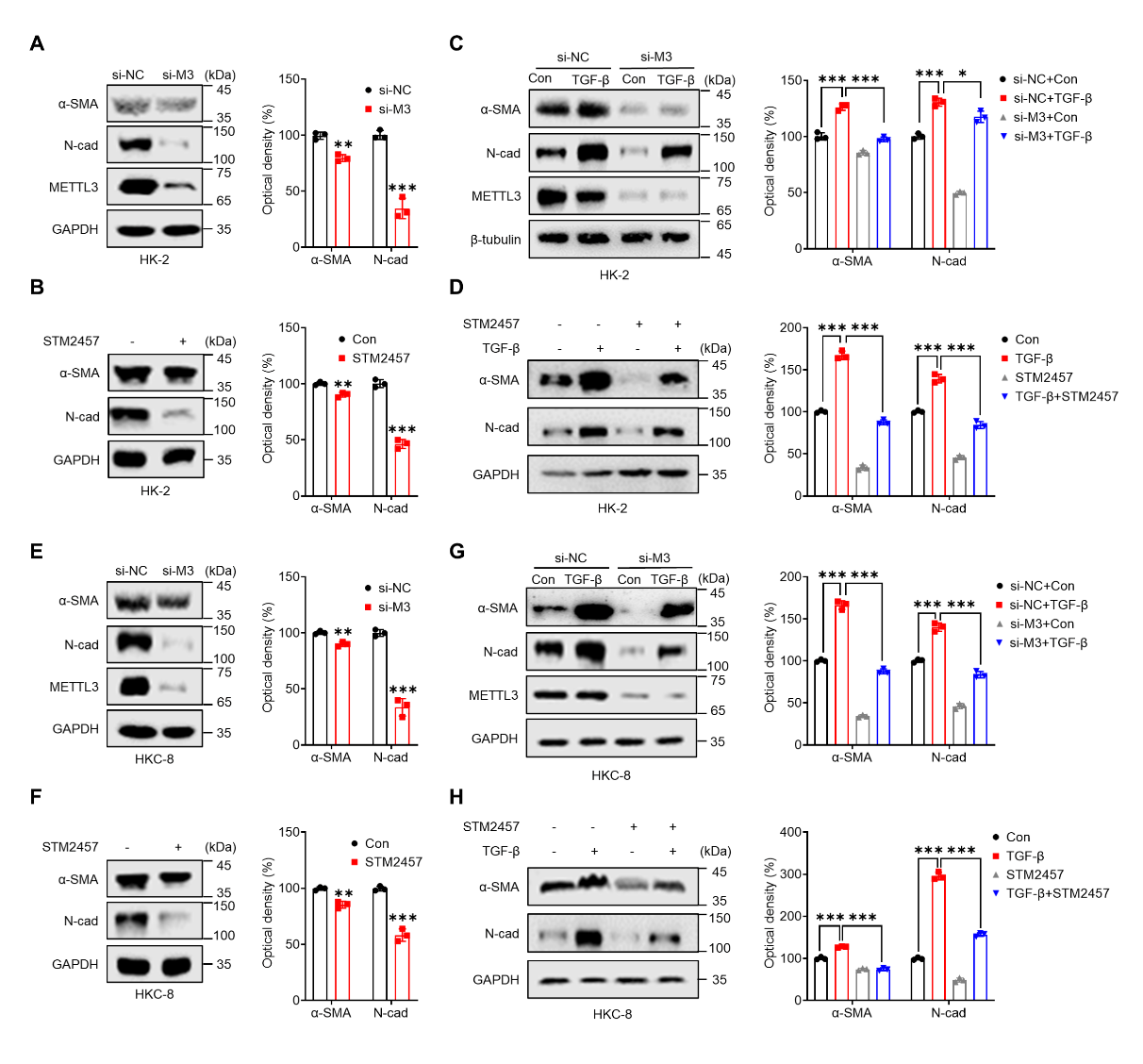


**Supplementary Figure3. Suppression of METTL3 inhibited EMT of renal tubular cells**

1. HK-2 cells were transfected with si-control (si-NC) or si-METTL3 (si-M3) for 48 h, and the protein expression levels of α-SMA and N-Cad were measured by western blot analysis (left) and quantified (right).
2. In HK-2 cells treated with or without STM2457 for 48 h, the protein levels of α-SMA and N-Cad were measured by western blot analysis (left) and quantified (right).
3. HK-2 cells were transfected with si-NC or si-M3 for 24 h and then further treated with or without 10 ng/ml TGF-β for 3 days. The protein levels of α-SMA and N-Cad were measured by western blot analysis (left) and quantitatively analyzed (right).
4. HK-2 cells were pretreated with or without STM2457 and then further treated with 10 ng/ml TGF-β for 3 days. The protein levels of α-SMA and N-Cad were measured by western blot analysis (left) and quantified (right).
5. The protein levels of α-SMA、N-Cad and ZEB2 in HKC-8 si-control(si-NC) and HKC-8 si-METTL3(si-M3) cells were measured by western blot analysis(left) and quantitatively analyzed (right).
6. HKC-8 cells treated with or without STM2457 for 48h, the protein levels of α-SMA、N-Cad and ZEB2 were measured by and western blot analysis (left) and quantitatively analyzed (right).
7. HKC-8 cells were transfected with siRNA for negative control (si-NC) or siRNA for METTL3(si-M3) for 24 h, and then further treated with or without 10 ng/ml TGF-β for 3 days, protein levels of ZEB2, α-SMA and N-Cad were measured by western blot analysis (left) and quantitatively analyzed (right).
8. HKC-8 cells were pretreated with or without STM2457 and then further treated with 10 ng/ml TGF-β for 3 days, protein levels of α-SMA and N-Cad were measured by western blot analysis (left) and quantitatively analyzed (right).

Data are presented as means ± SD from three independent experiments. *p < 0.05, **p < 0.01, ***p < 0.001, NS, no significant, by Student’s t test


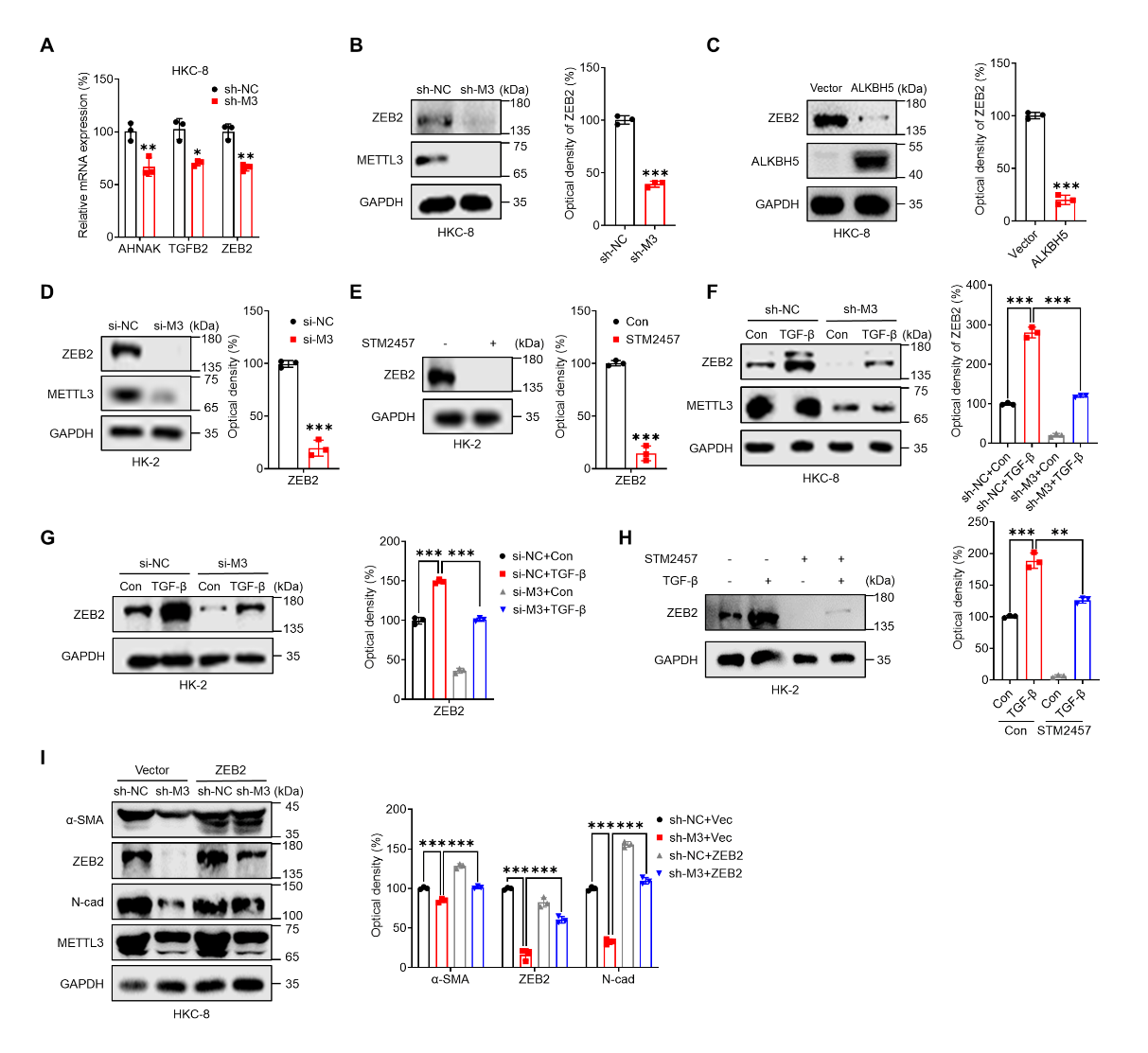


**Supplementary Figure4. ZEB2 was involved in m6A-regulated EMT in renal tubular epithelial cells**

1. The mRNA levels of AHNAK、TGFB2 and ZEB2 in sh-control or sh-METTL3 HKC-8 cells were measured by qRT-PCR.
2. The protein levels of ZEB2 in sh-control or sh-METTL3 HKC-8 cells were determined by western blot analysis (left) and quantitatively analyzed (right).
3. HKC-8 cells were transfected with pcDNA (vector) or pcDNA/ALKBH5 for 48 h, the protein expressions of ZEB2 were measured by western blot analysis (left) and quantitatively analyzed (right).
4. The protein levels of ZEB2 in HK-2 si-control(si-NC) and HKC-8 si-METTL3(si-M3) cells were measured by western blot analysis(left) and quantitatively analyzed (right).
5. HK-2 cells treated with or without STM2457 for 48h, the protein levels of ZEB2 were measured by and western blot analysis (left) and quantitatively analyzed (right).
6. sh-control or sh-METTL3 HKC-8 cells were treated with or without 10 ng/ml TGF-β for 3 days, protein levels of ZEB2 were measured by western blot analysis (left) and quantitatively analyzed (right).
7. HK-2 cells were transfected with negative control siRNA (si-NC) or METTL3 siRNA (si-M3) for 24 h and then further treated with or without 10 ng/ml TGF-β for 3 days. ZEB2 protein levels were measured by western blot analysis (left) and quantitatively analyzed (right).
8. HK-2 cells were pretreated with or without STM2457 and then further treated with or without 10 ng/ml TGF-β for 3 days. ZEB2 protein levels were measured by western blot analysis (left) and quantified (right).
9. sh-control or sh-METTL3 HKC-8 cells were transfected with or without pcDNA/ZEB2 for 48 h, The protein levels of ZEB2, α-SMA and N-Cad were measured by western blot analysis (left) and quantitatively analyzed (right).

Data are presented as means ± SD from three independent experiments. *p < 0.05, **p < 0.01, ***p < 0.001, NS, no significant, by Student’s t test


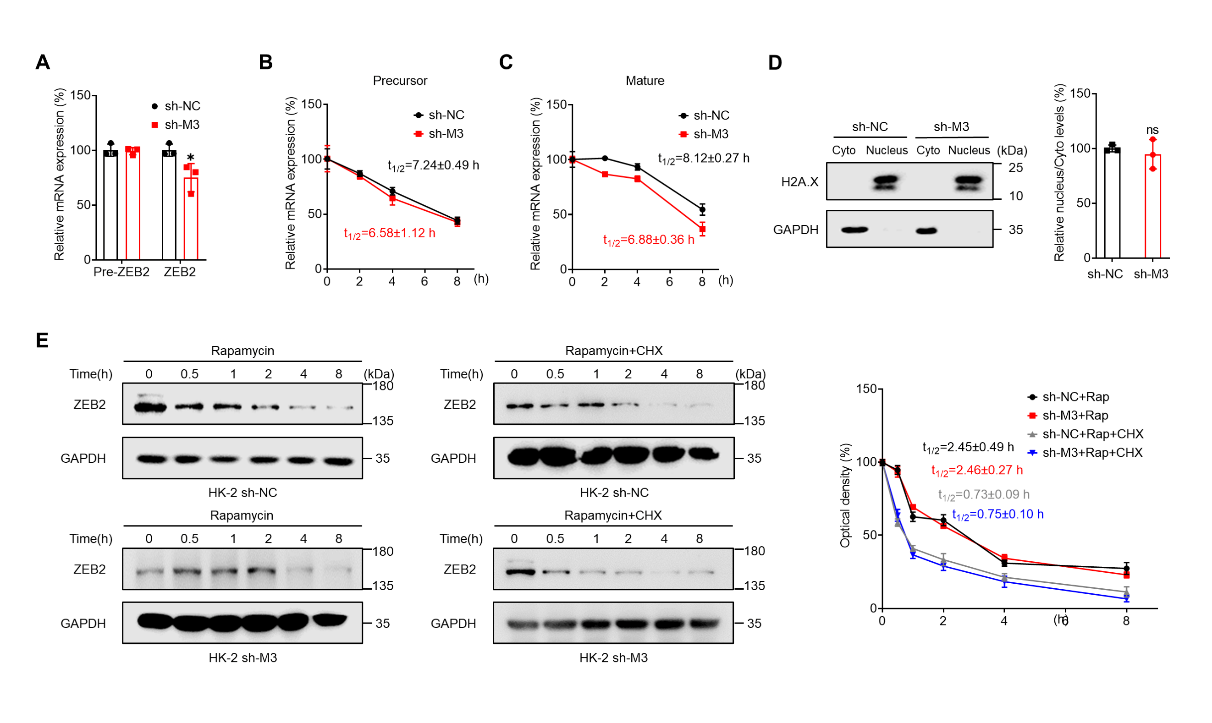


**Supplementary Figure5. m6A regulated ZEB2 mRNA stability and translation in renal tubular epithelial cells**

1. Precursor and mature mRNA of ZEB2 in sh-control or sh-METTL3 HKC-8 cells were measured by qRT-PCR.
2. sh-control or sh-METTL3 HKC-8 cells were pretreated with Act-D for the indicated times, the precursor ZEB2 mRNA were analyzed at indicated times.
3. sh-control or sh-METTL3 HKC-8 cells were pretreated with Act-D for the indicated times, the mature ZEB2 mRNA were analyzed at indicated times.
4. The relative levels of nuclear versus cytoplasmic ZEB2 mRNA in sh-control or sh-METTL3 HK-2 cells.
5. sh-control or sh-METTL3 HK-2 cells were treated with 50 nM rapamycin ±cycloheximide (CHX, 100 μg/ml), the protein levels of ZEB2 were determined by western blot analysis (left) and quantitatively analyzed (right).

Data are presented as means ± SD from three independent experiments. *p < 0.05, **p < 0.01, ***p < 0.001, NS, no significant, by Student’s t test


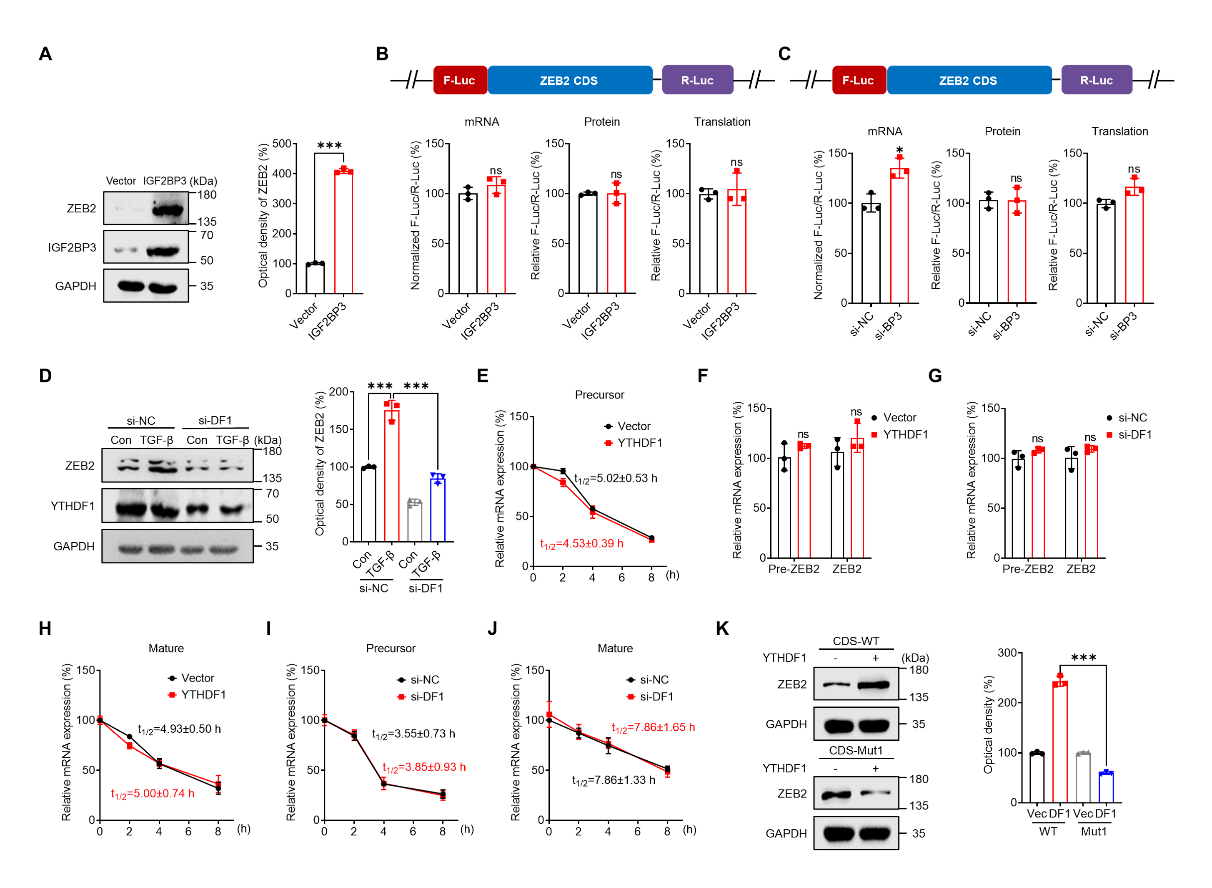


**Supplementary Figure6. Factors involved in the m6A-regulated expression of ZEB2**

1. HK-2 cells were transfected with vector or IGF2BP3 construct for 48 h, the expression of ZEB2 was checked by western blot analysis (left) and quantitatively analyzed (right).
2. HK-2 cells were transfected with vector or IGF2BP3 construct for 24 h, and then further treated with pmirGLO-ZEB2 reporter for 24 h, and the translation efficiency of ZEB2 is defined as the quotient of reporter protein production (F-luc/R-luc) divided by mRNA abundance.
3. HK-2 cells were transfected with siRNA for negative control (si-NC) or siRNA for IGF2BP3(si-BP3) for 24 h, and then further treated with pmirGLO-ZEB2 reporter for 24 h, and the translation efficiency of ZEB2 is defined as the quotient of reporter protein production (F-luc/R-luc) divided by mRNA abundance.
4. After being pre-transfected with siRNA for negative control (si-NC) or siRNA for YTHDF1(si-DF1) for 24h, HK-2 cells were further treated with or without 10 ng/ml TGF-β for 48 h, the expression of ZEB2 was checked by western blot analysis (left) and quantitatively analyzed (right).
5. HK-2 cells were transfected with the vector or the YTHDF1 construct for 24 h, and the levels of the precursor and mature ZEB2 mRNAs were measured via qRT‒PCR.
6. HK-2 cells were transfected with siRNA for the negative control (si-NC) or siRNA for YTHDF1 (si-DF1) for 24 h, and the precursor and mature mRNAs of ZEB2 were measured by qRT‒PCR.
7. HK-2 cells were transfected with vector, YTHDF1 construct, pcDNA-ZEB2-CDS-WT, and pcDNA-ZEB2-CDS-Mut1 for 24 h, and then further treated with Act-D for the indicated times. The precursor ZEB2 mRNA were analyzed at indicated times.
8. HK-2 cells were transfected with vector, YTHDF1 construct, pcDNA-ZEB2-CDS-WT, and pcDNA-ZEB2-CDS-Mut1 for 24 h, and then further treated with Act-D for the indicated times. The mature ZEB2 mRNA were analyzed at indicated times.
9. HK-2 cells were transfected with siRNA for negative control (si-NC), siRNA for YTHDF1(si-DF1), pcDNA-ZEB2-CDS-WT, and pcDNA-ZEB2-CDS-Mut1 for 24 h, and then further treated with Act-D for the indicated times. The precursor ZEB2 mRNA were analyzed at indicated times.
10. HK-2 cells were transfected with siRNA for negative control (si-NC), siRNA for YTHDF1(si-DF1), pcDNA-ZEB2-CDS-WT, and pcDNA-ZEB2-CDS-Mut1 for 24 h, and then further treated with Act-D for the indicated times. The mature ZEB2 mRNA were analyzed at indicated times.
11. HK-2 cells were transfected with vector, YTHDF1 construct, pcDNA-ZEB2-CDS-WT, and pcDNA-ZEB2-CDS-Mut1 for 48 h, the expression of ZEB2 was checked by western blot analysis (left) and quantitatively analyzed (right).

Data are presented as means ± SD from three independent experiments. *p < 0.05, **p < 0.01, ***p < 0.001, NS, no significant, by Student’s t test


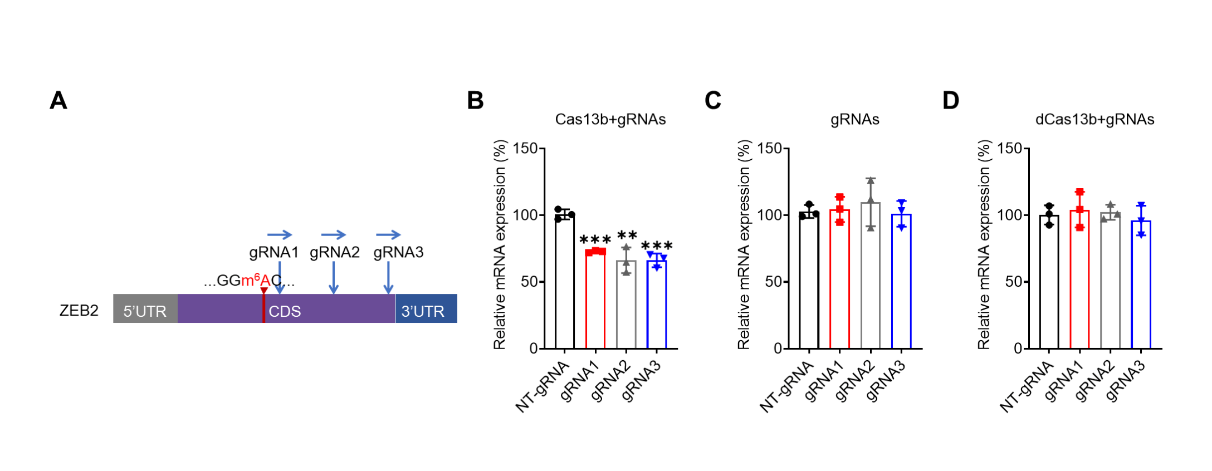


**Supplementary Figure7. Targeting m6A of ZEB2 mRNA by dm6ACRISPR re-programed EMT of renal tubular cells**

1. Schematic representation of the positions of the m6A site within ZEB2 mRNA and the regions targeted by the three gRNAs.
2. The mRNA expression of ZEB2 in HK-2 cells transfected with Cas13b combined with NT-gRNA or gRNA1/2/3, respectively, for 24 h.
3. The mRNA expression of ZEB2 in HK-2 cells transfected with gRNA alone for 24h.
4. The mRNA expression of ZEB2 in HK-2 cells transfected with dCas13b combined with NT-gRNA or gRNA1/2/3, respectively, for 24 h.

Data are presented as means ± SD from three independent experiments. *p < 0.05, **p < 0.01, ***p < 0.001, NS, no significant, by Student’s t test


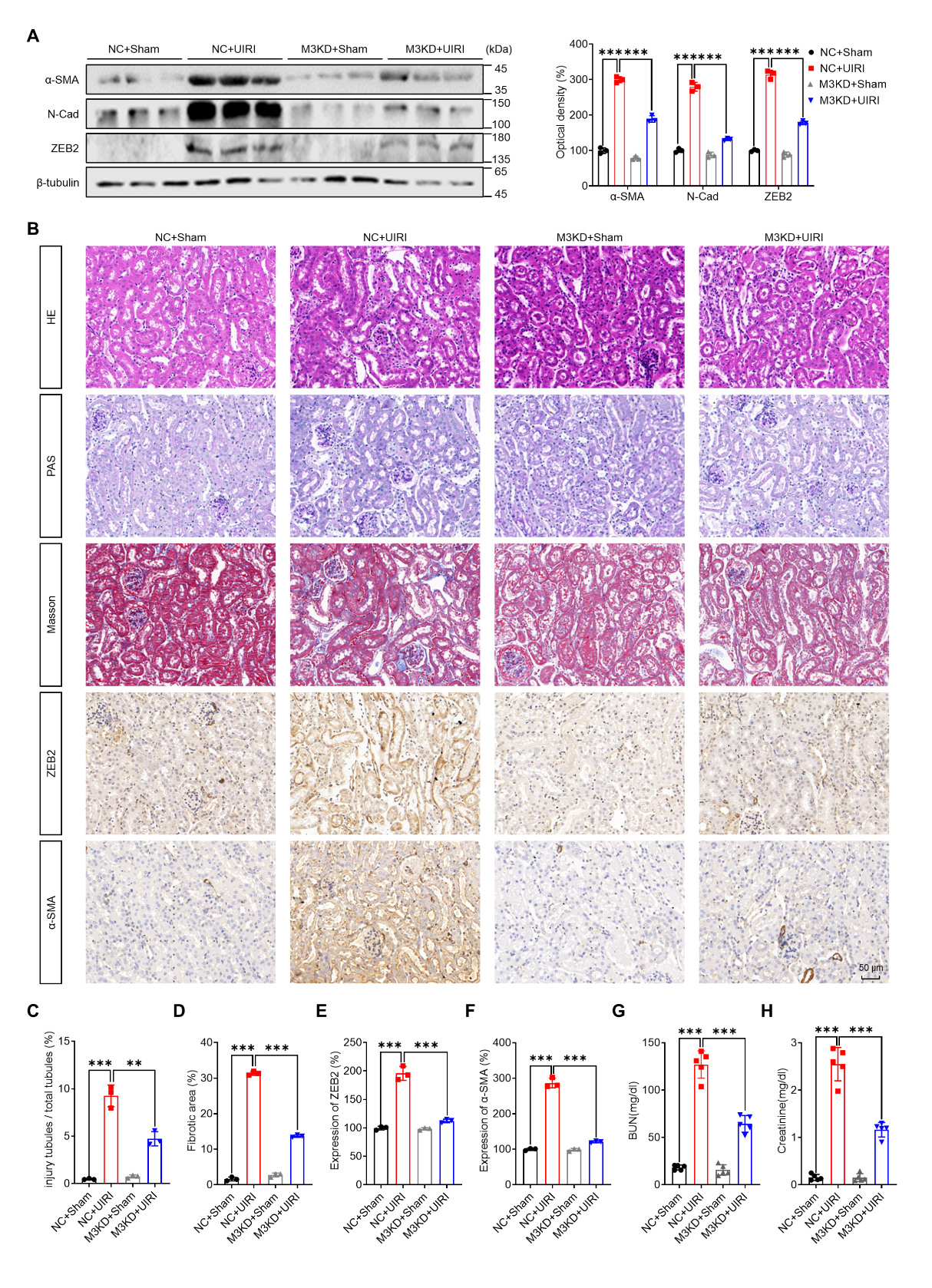


**Supplementary Figure8. m6A modification regulated renal fibrosis *in vivo***

1. The protein of α-SMA、N-Cad and ZEB2 in the UIRI model with or without knockdown of METTL3 were measured by western blot analysis (left) and quantitatively analyzed (right).

(B~F) HE、PAS、Masson and IHC staining in the UIRI model with or without knockdown of METTL3(B) were quantitatively analyzed(C~F).

1. Serum BUN in mice with in the UIRI model with or without knockdown of METTL3.
2. Serum Cr in mice with in the UIRI model with or without knockdown of METTL3.

Data are presented as means ± SD from three independent experiments. *p < 0.05, **p < 0.01, ***p < 0.001, NS, no significant, by Student’s t test
